# Marine nitrogen fixers mediate a low latitude pathway for atmospheric CO_2_ drawdown

**DOI:** 10.1038/s41467-019-12549-z

**Published:** 2019-10-10

**Authors:** Pearse J. Buchanan, Zanna Chase, Richard J. Matear, Steven J. Phipps, Nathaniel L. Bindoff

**Affiliations:** 1 Department of Earth, Ocean and Ecological Sciences, University of Liverpool, L69 3GP Liverpool, UK; 2 Institute for Marine and Antarctic Studies, University of Tasmania, Hobart, 7004 Australia; 3 CSIRO Oceans and Atmosphere, CSIRO Marine Laboratories, G.P.O Box, 1538 Hobart, Tasmania Australia; 4 ARC Centre of Excellence in Climate System Science, Hobart, Tasmania 7004 Australia; 5 ARC Centre of Excellence in Climate Extremes, Hobart, Tasmania 7004 Australia; 6 Antarctic Climate and Ecosystems Cooperative Research Centre, Hobart, Tasmania 7004 Australia

**Keywords:** Carbon cycle, Carbon cycle, Element cycles, Marine chemistry

## Abstract

Roughly a third (~30 ppm) of the carbon dioxide (CO_2_) that entered the ocean during ice ages is attributed to biological mechanisms. A leading hypothesis for the biological drawdown of CO_2_ is iron (Fe) fertilisation of the high latitudes, but modelling efforts attribute at most 10 ppm to this mechanism, leaving ~20 ppm unexplained. We show that an Fe-induced stimulation of dinitrogen (N_2_) fixation can induce a low latitude drawdown of 7–16 ppm CO_2_. This mechanism involves a closer coupling between N_2_ fixers and denitrifiers that alleviates widespread nitrate limitation. Consequently, phosphate utilisation and carbon export increase near upwelling zones, causing deoxygenation and deeper carbon injection. Furthermore, this low latitude mechanism reproduces the regional patterns of organic *δ*^15^N deposited in glacial sediments. The positive response of marine N_2_ fixation to dusty ice age conditions, first proposed twenty years ago, therefore compliments high latitude changes to amplify CO_2_ drawdown.

## Abstract (web summary)

Iron fertilisation of the high latitude oceans is a well-established biological mechanism to explain the ice age drawdown of atmospheric CO_2_, yet modelling has so far struggled to account for a sufficient drawdown via this mechanism. Here, the authors propose that N_2_ fixers, which inhabit the lower latitude ocean, made a significant contribution to CO_2_ drawdown and so amplified the global response to iron fertilisation during ice ages.

## Introduction

As much as 30 ppm of the total glacial-interglacial difference in atmospheric CO_2_ is attributed to marine biological mechanisms^1^. The most prominent biological mechanism is the fertilisation of Fe-limited high latitude regions, namely the Southern Ocean^2^ and subarctic Pacific^3^, with dust-borne Fe under dusty glacial conditions^4,5^. Today, phytoplankton that inhabit these high latitude regions are unable to consume all available macronutrients, which allows CO_2_ to escape to the atmosphere as deep waters mix into surface layers. Iron fertilisation of the high latitude glacial ocean therefore stands as a leading hypothesis to explain a more efficient biological carbon (C) pump and the associated drawdown of atmospheric CO_2_. Yet, modelling focussed on the high latitudes has sequestered less than 10 ppm of atmospheric CO_2_ via Fe fertilisation^5–7^ and indicates that additional biological mechanisms are required.

There are good reasons to accommodate the lower latitudes in our search for additional mechanisms. First, the region is enormous. Surface waters between 40°S and 40°N represent over two thirds of CO_2_ outgassing to the atmosphere^8^ and more than half of global C export^9,10^. Second, unconsumed phosphate (PO_4_) at concentrations in excess of 0.1 to 0.2 mmol m^−3^ exists in surface waters across the tropics, which is evidence for unrealised biological CO_2_ fixation. Third, tropical oceans produce organic matter that is enriched in C because tropical phytoplankton are adapted to fix more C per unit phosphorus (P) under P scarcity^11^. Fourth, oxygen-deficient waters in the tropical Pacific, Indian and Atlantic allow organic matter to sink deeper into the ocean interior^10,12,13^. If these mechanisms are combined, the co-occurrence of more complete PO_4_ utilisation and the production of C-enriched organic matter near to oxygen-deficient zones would constitute an effective pathway of CO_2_ drawdown.

Enabling greater PO_4_ utilisation and CO_2_ drawdown in the lower latitudes, however, requires simultaneously relieving Fe limitation in upwelling zones^14^, nitrate (NO_3_) limitation in the tropics^14,15^ and their co-limitation at the boundary of both regimes^16^. An aeolian Fe-induced stimulation of dinitrogen (N_2_) fixation is therefore an obvious candidate to alleviate low latitude nutrient limitation. Originally proposed by Falkowski^17^, this mechanism is now supported by many independent lines of evidence. N_2_ fixers are highly sensitive to the aeolian supply of Fe^18,19^, they represent up to half of primary production and C export in oligotrophic waters^20–24^, they are physiologically adapted to P scarcity^25,26^, produce organic matter that is enriched in C^27–29^, and previous modelling has demonstrated the potential of N_2_ fixation to draw CO_2_ into the ocean^30^. Dinitrogen fixation is also inextricably linked to suboxic zones (dissolved oxygen (O_2_) < 10 mmol m^−3^) where denitrification strips NO_3_ from the waters that upwell at the equator, creating a potential niche for N_2_ fixers across the wide expanse of the lower latitudes. The strength of N_2_ fixation, which strengthens PO_4_ utilisation, whole community C:P ratios and C export^20^, is thus tied to the strength of denitrification, which in turn strengthens N_2_ fixation.

In this study, we use an ocean model to demonstrate that aeolian Fe supply to the tropical oceans under glacial conditions^31,32^ relieves low latitude nutrient limitation^14–16^ by stimulating N_2_ fixation, which in turn drives PO_4_ consumption, suboxic zone expansion, the acceleration of the nitrogen (N) cycle and a more efficient C export to the interior ocean. Furthermore, we estimate the contribution of this mechanism to CO_2_ drawdown and reveal evidence of its existence within glacial-interglacial sedimentary records of N isotopes (*δ*^15^N_*org*_).

## Results

### A low latitude pathway

Inspired by these insights, we undertook multi-millennial simulations using a global ocean biogeochemical model to explore the link between Fe fertilisation, N_2_ fixation and CO_2_ drawdown. The ocean biogeochemical model is part of the Commonwealth Scientific and Industrial Research Organisation (CSIRO) Mark 3L—Carbon of the Ocean, Atmosphere and Land (Mk3L-COAL)^33^. The model is designed for long-term, global oceanographic studies. It resolves multi-millennial timescales and so produces equilibrium circulation states under a given set of atmospheric conditions. It is equipped with prognostic C, PO_4_, NO_3_, ^15^NO_3_, and Fe cycles^34^ (see Methods), and includes a dynamic ecosystem component where phytoplankton alter their nutrient requirements, stoichiometry and remineralisation rates according to their environment^33^ (Supplementary Fig. 1). We increased the supply of aeolian Fe to the ocean model from its modern^35^ to glacial rate^5^ (see Methods; Supplementary Fig. 2) under preindustrial physical conditions (Mk3L^*mild*^ state in Table 1; Supplementary Note 1; Supplementary Figs. 3 and 4; Supplementary Table 1) with an atmospheric CO_2_ held at 280 ppm, and assessed changes to elemental cycling. To isolate the response of the lower latitudes, we nudged subsurface Fe concentrations to 0.6 μmol m^−3^ on a yearly timescale, which ensured that Fe was near non-limiting in regions of strong mixing, like the Southern Ocean and subarctic Pacific.

**Table 1:** Global properties of the four ocean states

Variable	Units	GFDL^*warm*^	Mk3L^*mild*^	HadGEM^*cool*^	Mk3L^*cold*^
Temp^a^	(°C)	5.3	3.9	3.5	1.4
Sal^a^	(psu)	34.72	34.50	34.38	35.49
*δ*^14^C^*a*^	(‰)	−143.9	−151.5	−158.4	−184.2
Surface PO_4_^a^	(mmol m^−3^)	0.45	0.36	0.30	0.28
O_2_^a^ (AOU^a^)	(mmol m^−3^)	172 (136)	188 (134)	222 (103)	243 (95)
Suboxia^b^	(% ocean)	3.6	2.7	2.1	1.6
NO_3_^a^	(mmol m^−3^)	22.1	22.4	24.0	28.9
Ψ_*AABW*_^c^	(Sv)	7.2	11.5	11.4	39.0
Ψ_*NADW*_^d^	(Sv)	20.3	18.4	13.0	13.0
$\frac{{\partial \rho }}{{\partial z}}$ (0–500 m)^e^	(kg m^−3^)	5.41 × 10^−3^	5.06 × 10^−3^	4.91 × 10^−3^	6.39 × 10^−3^
$\frac{{\partial \rho }}{{\partial z}}$ (1–2 km)^e^	(kg m^−3^)	0.51 × 10^−3^	0.45 × 10^−3^	0.65 × 10^−3^	1.62 × 10^−3^

^a^Global mean values. All other properties are integrated totals

^b^Suboxia refers to waters < 10 mmol O_2_ m^−3^

^c^Formation rate of Antarctic Bottom Water (AABW)

^d^Formation rate of North Atlantic Deep Water (NADW)

^e^A measure of density change, and hence stratification, averaged over a depth interval

**Fig. 1:**
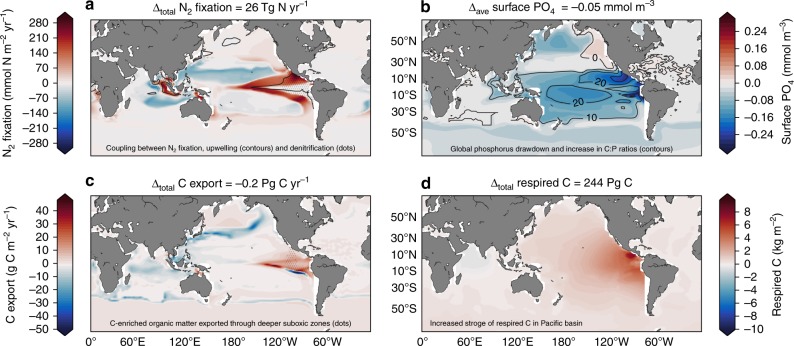
Biogeochemical response to an Fe-induced stimulation of N_2_ fixers. Change in **a** N_2_ fixation rate, **b** surface PO_4_ concentration, **c** carbon export rate and **d** total respired carbon through the water column. The changes shown in both the coloured shading and the headings above each panel show the effect of increasing Fe deposition from the modern flux to the glacial flux^5^. The headings describe the integrated change (Δ_*total*_) or the average change (Δ_*ave*_) of each property, calculated as the volume or area weighted sum/average of each property. Dots in (**a**) represent active water column denitrification. Contours in (**a**) define upwelling where ideal water age >25 years at 80 metres depth. Contours in (**b**) are the change in C:P ratios of exported organic matter. Dots (dashes) in (**c**) define a vertical expansion (shrinking) of suboxia >500 metres

**Fig. 2:**
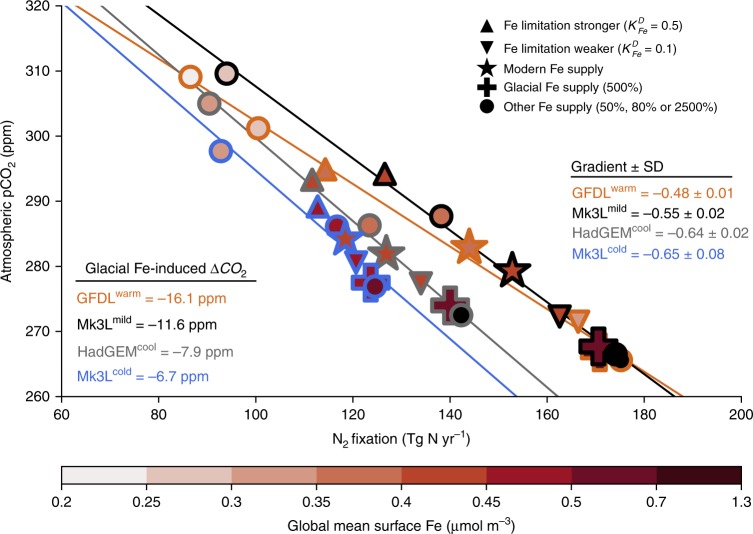
N_2_ fixation and its relationship with atmospheric CO_2_. The atmospheric reservoir of CO_2_ was made responsive to oceanic uptake and release of C by the ocean (see methods). Coloured edges of markers correspond to ocean states as described in the figure legend. Star markers represent oceans with carbon cycles equilibrated to the modern Fe deposition^36^ under atmospheric CO_2_ of −280 ppm. The plus symbol in each ocean state represents the change due to a glacial increase in Fe supply^6^, which represents a global integrated increase of 500% over the modern supply but is regionally variable (Supplementary Fig. 2). Triangles represent changes in the half-saturation constant for Fe limitation of N_2_ fixers, emulating changes in Fe supply to only N_2_ fixers. Note the diminishing gains in CO_2_ drawdown between 500 and 2500% iron deposition scenarios, consistent with Fe-saturation and PO_4_ limitation. Regression lines and their slopes (gradient ± standard deviation (SD)) represent the linear relationship between N_2_ fixation and atmospheric CO_2_, with a multi-ocean mean of 0.58 ± 0.03 ppm CO_2_ sequestered for every additional Tg N yr^−1^. Source data are provided in the source data file

The glacial aeolian Fe supply increased the global rate of N_2_ fixation by 26 Tg N yr^−1^ and caused a large-scale change in its distribution (Fig. 1a). Dinitrogen fixers exhibited a closer coupling to regions of strong upwelling in the tropics (solid contour in Fig. 1a) that are co-located with areas of denitrification (dots in Fig. 1a). The greatest changes were observed in the Pacific. Dinitrogen fixation decreased in the Northwest Pacific and increased in the Eastern Tropical Pacific, which hosted low rates of N_2_ fixation under modern Fe supply (Supplementary Fig. 5a). As a result, surface PO_4_ was reduced throughout the tropical Pacific by between 0.1 and 0.2 mmol m^−3^ (Fig. 1b; Supplementary Fig. 5b). Pacific PO_4_ utilisation increased the C:P ratio of exported organic matter by an average of ~14 units (contours in Fig. 1b), which elevated local C export (Fig. 1c; Supplementary Fig. 5c), caused a vertical expansion of suboxia (dots in Fig. 1c), and enabled the permanent accumulation of 244 Pg of respired C in the eastern Pacific (Fig. 1d; Supplementary Fig. 5d). As a result, 11.6 ppm of CO_2_ was permanently sequestered (see “Quantifying CO_2_ drawdown” and Fig. 2).

Dust-borne Fe fertilisation therefore involved a set of biogeochemical feedbacks, not possible by increasing the NO_3_ inventory (Supplementary Note 2; Supplementary Fig. 6), that enabled CO_2_ drawdown. Tropical upwelling zones are highly productive regions that drive strong subsurface O_2_ depletion, which in turn stimulates denitrification and strips upwelling waters of NO_3_. Consequently, the tropical Pacific hosts low NO_3_:PO_4_ ratios (Supplementary Fig. 7a), which provides a competitive niche for N_2_ fixers. Today, the low supply of aeolian Fe to the tropical Pacific^31,35^ prevents N_2_ fixers from inhabiting this niche^24^, and allows excess, unconsumed PO_4_ (>0.2 mmol m^−3^) to spill 10–15° either side of the equator^36^ (Supplementary Fig. 7b). In contrast, the glacial Fe supply allowed N_2_ fixers to inhabit the low NO_3_:PO_4_ waters at the boundary to upwelling zones where local Fe-N co-limitation prevails today^14,16^. This shift in N_2_ fixation initiated strong biogeochemical feedbacks that encouraged PO_4_ utilisation, C export, suboxic expansion, denitrification, and a local NO_3_ supply via N_2_ fixation. Two consequences of this simulated feedback, the local increase in C export and a vertical expansion of suboxia, enabled the transfer of large amounts of C-rich organic matter deep within the interior of the Pacific basin (Fig. 1d).

### Quantifying CO_2_ drawdown

We sought to quantify the sensitivity of atmospheric CO_2_ drawdown to the physical conditions of the ocean, as glacial conditions were distinct from pre-industrial^37^. We produced four different ocean states that can be considered broadly representative of glacial-interglacial conditions, encompassing warm to cold, well-mixed to stratified, and thus interglacial to glacial (Table 1; Supplementary Fig. 8). Dust-borne Fe supply was varied to 50, 80, 100, 500% (glacial) and 2500% of the modern rate^35^ over these four ocean states (see Methods) to fully encompass the glacial-interglacial range in conditions. Both high Fe deposition scenarios (500 and 2500%) are based on the climatology of Lambert^5^, meaning that the delivery of Fe is not uniformly greater everywhere (Supplementary Fig. 2). The tropical Pacific, for instance, receives roughly 2-fold more Fe than under modern conditions consistent with recent estimates^31,32^. In addition, we increased and decreased the Fe requirements of N_2_ fixers without varying aeolian Fe deposition (See methods), which emulated variations in Fe supply but only to N_2_ fixers. If similar changes occurred via both methods, then N_2_ fixation could be considered the primary driver of CO_2_ drawdown.

The ocean states were GFDL^*warm*^, Mk3L^*mild*^ (control state used previously), HadGEM^*cool*^ and Mk3L^*cold*^. GFDL^*warm*^ was the warmest, youngest (see *δ*^14^C), most deoxygenated, NO_3_-deplete and PO_4_-rich ocean, with a rapid overturning circulation dominated by the upper cell. Mk3L^*mild*^ and HadGEM^*cool*^ were cooler, fresher and formed greater quantities of Antarctic Bottom Water than GFDL^*warm*^. The key difference between Mk3L^*mild*^ and HadGEM^*cool*^ was the rate of North Atlantic Deep Water formation, which was stronger for Mk3L^*mild*^ and elevated surface PO_4_, C export, O_2_ consumption and denitrification rates. Mk3L^*cold*^ represented full glacial conditions. It was the coldest, saltiest, and oldest ocean state, featuring strong vertical density gradients that restricted PO_4_ supply and a greatly expanded lower overturning cell consistent with glacial conditions^38^.

An increase in Fe supply drew between 6.7 and 16 ppm of atmospheric CO_2_ into the ocean (compare star and plus symbols in Fig. 2). Different ocean states (colours in Fig. 2) therefore absorbed different quantities of CO_2_. However, all states developed a positive, linear relationship between N_2_ fixation and CO_2_ drawdown (coloured lines in Fig. 2). A consistent relationship between N_2_ fixation and CO_2_ drawdown suggested that all states absorbed atmospheric CO_2_ via the same low latitude pathway described in previously. Approximately 0.58 ± 0.03 ppm of CO_2_ was absorbed by the ocean for every additional Teragram of N fixed per year (Tg N yr^−1^). The linear relationship was generated as N_2_ fixation responded to variations in Fe supply (circles) and as N_2_ fixation was made more or less sensitive to the modern supply of Fe (triangles). Similar responses occurred via both methods (altered Fe deposition and Fe requirements) and strongly implicated N_2_ fixation as the driver of CO_2_ drawdown.

The sensitivity of each ocean state to changes in N_2_ fixation was fundamentally linked to the strength of equatorial upwelling. The greatest sensitivity was found in GFDL^*warm*^, which featured strong upwelling, and therefore high surface PO_4_ and large suboxic zones (Table 1). Consequently, large regions of the tropical ocean were low in NO_3_:PO_4_, which enabled large gains in N_2_ fixation (88 Tg N yr^−1^) and CO_2_ drawdown (43 ppm) as Fe supply increased from 50 to 2500% of its modern rate. In contrast, Mk3L^*cold*^ featured the weakest rates of upwelling, lowest surface concentrations of PO_4_, the smallest suboxic zones (Table 1), and thus the weakest sensitivity. Phosphate availability therefore emerged as the ultimate control on biological CO_2_ drawdown by setting N_2_ fixation potential, while Fe supply modulated the extent to which this potential was realised.

### A central role for N_2_ fixers

The previous experiments showed that N_2_ fixers responded to Fe addition leading to reduced atmospheric CO_2_. To elucidate the mechanisms through which this occurred we considered several additional experiments with the Mk3L^*mild*^ ocean state subject to variations in aeolian Fe supply. First, we removed N_2_ fixers and denitrification completely, thereby holding the NO_3_ reservoir constant. Second, we reinstated N_2_ fixers (NO_3_ supply) and a marine N cycle (active denitrification), but removed their C export by setting their C:P ratio equal to zero. Third, we decreased their C:P ratio to 165:1, half its default of 331:1^39^. Fourth, we reinstated their default C:P ratio of 331:1, but increased their PO_4_ half-saturation coefficient $\left( {K_{PO_4}^D} \right)$ to 0.1 mmol m^−3^, which is the same as the general phytoplankton group and so removed their competitive advantage for PO_4_.

**Fig. 3:**
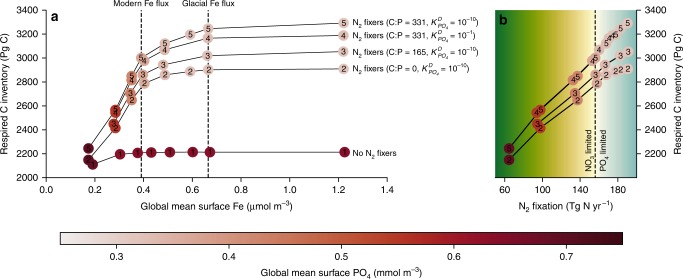
How N_2_ fixation enables CO_2_ storage in the ocean. The response of the respired C inventory to aeolian Fe deposition experiments for five different representations of N_2_ fixers, with **a** demonstrating the relationship between respired C and Fe availability and **b** demonstrating the relationship between respired C and N_2_ fixation. The marker numbers refer to how N_2_ fixers are represented. Ones: no N_2_ fixers and no active N cycle. Twos: N_2_ fixers and N cycle (i.e., denitrification) reinstated but no N_2_ fixer carbon export (C:P = 0:1). Threes: N_2_ fixers C:P ratio increased to 165:1, half its default value. Fours: N_2_ fixers PO_4_ limitation enforced by increasing their half saturation coefficient $\left( {K_{PO_4}^D} \right)$ from 10^−10^ to 0.1 mmol m^−3^. Fives: N_2_ fixers with default parameterisation. Colour shading of the markers indicates the mean surface PO_4_ concentration of the experiment. Background shading in **b** is a qualitative indicator of the transition from NO_3_ to PO_4_ limitation, where dark green to yellow indicates NO_3_ limitation and light blue indicates PO_4_ limitation. Source data are provided in the source data file

These experiments revealed that N_2_ fixers were essential for C accumulation via the low latitudes. If N_2_ fixers were removed and the NO_3_ reservoir remained constant, greater Fe supply did not cause respired C storage (ones in Fig. 3a). Insensitivity to Fe supply was due to widespread NO_3_ limitation of lower latitude ecosystems^15^. The simple addition of N_2_ fixers without changes in Fe increased NO_3_ supply to surface waters (Supplementary Fig. 9) and increased PO_4_ utilisation between 40°S and 40°N by 7%. Dinitrogen fixers were, therefore, able to provide significant gains to the oceanic C store over millennia, which extends insights of in situ studies^20–23^ and prior modelling^30^ to the scale of the glacial cycles, as originally proposed by Falkowski^17^.

Dinitrogen fixers were therefore essential for oceanic C storage for the simple reason that they supplied fixed N to the upper ocean. Fixed N supply was responsible for 70% of the C gains (~5–11 ppm) and responsible for the increase in PO_4_ utilisation as Fe supply increased (compare ones and twos in Fig. 3a). Included within this C storage was the increase in C:P ratios of Pacific Ocean phytoplankton as PO_4_ concentrations declined (see Fig. 1b). The final 30% (~2–5 ppm CO_2_) of additional C gain was mostly due to export of N_2_ fixer’s C-rich organic matter as the ocean became PO_4_-limited (compare ones, threes and fives in Fig. 3a), while their efficient utilisation of PO_4_ provided a small benefit to oceanic C storage (compare fours and fives in Fig. 3a). As N_2_ fixers already inhabit a niche of low NO_3_:PO_4_^20^, they are already at a competitive advantage over non-N_2_ fixing phytoplankton for available PO_4_.

The linear relationship between N_2_ fixation and CO_2_ drawdown, which was robust across different ocean states (Fig. 2), was therefore built on two phases. The first phase (green-yellow shading in Fig. 3b) occurred in a NO_3_-limited ocean, where N_2_ fixation increased the supply of NO_3_ to surface communities and thereby allowed excess PO_4_ to be consumed. The second phase (light blue shading in Fig. 3b) occurred in a PO_4_-limited ocean. As N_2_ fixers consumed proportionally more of the remaining PO_4_, their C export became more important for overall C export (compare ones, threes and fours/fives Fig. 3b). The slope of the linear relationship presented in Fig. 2, therefore, rested on a C:P ratio of N_2_ fixer organic matter equal to 331:1^39^. While there is significant variation around this number, under PO_4_-limiting conditions the C:P ratio tends to increase, exceeding 500:1 among *Trichodesmium* species^28^. Therefore, the C:P of N_2_ fixers could rise as PO_4_-limiting conditions develop and steepen the linear relationship to enable greater CO_2_ drawdown above that suggested here.

### Glacial *δ*^15^N records

To test our proposed mechanism of low latitude CO_2_ drawdown against observations, we simulated the response of the isotopic composition of organic N (*δ*^15^N_*org*_) to a glacial increase in Fe supply, and compared this response to a global compilation of glacial *δ*^15^N records (Supplementary Data 1). These experiments were completed within each ocean state presented in Table 1, so as to isolate the effect of Fe fertilisation from the effects of physical changes. In the following, we discuss the response using the Mk3L^*mild*^ ocean state, but each ocean state gave a similar response (Supplementary Fig. 10).

**Fig. 4:**
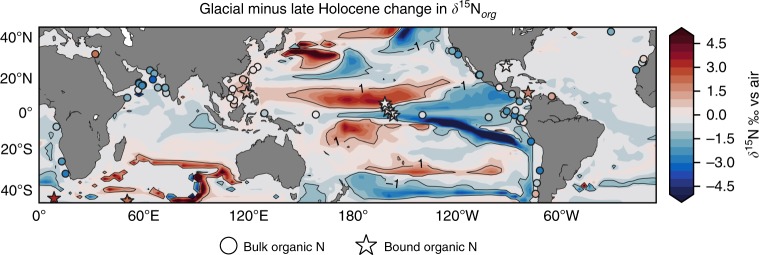
Seeking evidence from sedimentary *δ*^15^N_*org*_ records. Shading depicts the simulated change in *δ*^15^N of organic matter as a result of increasing aeolian Fe supply from the modern to a glacial rate. Solid and dashed contour lines mark simulated differences of 1 and −1. Circles mark locations of sediment cores where bulk organic matter was analysed for *δ*^15^N, while stars mark locations where either foraminifera- or diatom-bound *δ*^15^N was measured. The colour of the markers is an estimate of the glacial (Last Glacial Maximum: 20,000–26,000 BCE) minus interglacial (Late Holocene: 0–5000 BCE) difference in *δ*^15^N_*org*_, with red colours representing higher values and blues representing lower values in the glacial ocean. Sedimentary record data are provided in Supplementary Data 1

An Fe-induced coupling of N_2_ fixers to the upwelling zones of the eastern tropical Pacific increased *δ*^15^N_*org*_ in the west and decreased it in the east, which broadly reproduced patterns of glacial-interglacial change throughout the Pacific basin (Fig. 4). The increase in the western part of the basin was due to local decreases in N_2_ fixation and sedimentary denitrification, both of which lower *δ*^15^N. Our simulation of higher *δ*^15^N_*org*_ in the west Pacific, therefore, supports the interpretation of a recent foraminifera-bound record in the South China Sea^40^ (star marker). However, the simulated decrease in the *δ*^15^N of the eastern Pacific was not caused by a decrease in water column denitrification as suggested by numerous studies since the seminal paper of Ganeshram et al.^41^. Instead, our simulated decrease in eastern *δ*^15^N_*org*_ was caused by increases in both sedimentary denitrification and N_2_ fixation (Fig. 1a).

However, poor agreement was found in other regions, namely in the tropical western Atlantic and Southern Ocean where an increase in *δ*^15^N_*org*_ was not simulated. In the west Atlantic, Straub et al.^42^ presented a compelling relationship between *δ*^15^N and orbital precession, leading the authors to surmise a dependence on the upwelling of PO_4_ via changes in the circulation. In the Southern Ocean, a glacial increase in *δ*^15^N in Subantarctic^2^ and Antarctic zones^43,44^ is explained by a weaker physical delivery of NO_3_ to the mixed layer combined with Fe fertilisation. We therefore expected and found no response in both regions in these experiments (Supplementary Fig. 10) because the only change was an increase in dust-borne Fe and the Southern Ocean was made insensitive to increases in Fe supply.

## Discussion

**Fig. 5:**
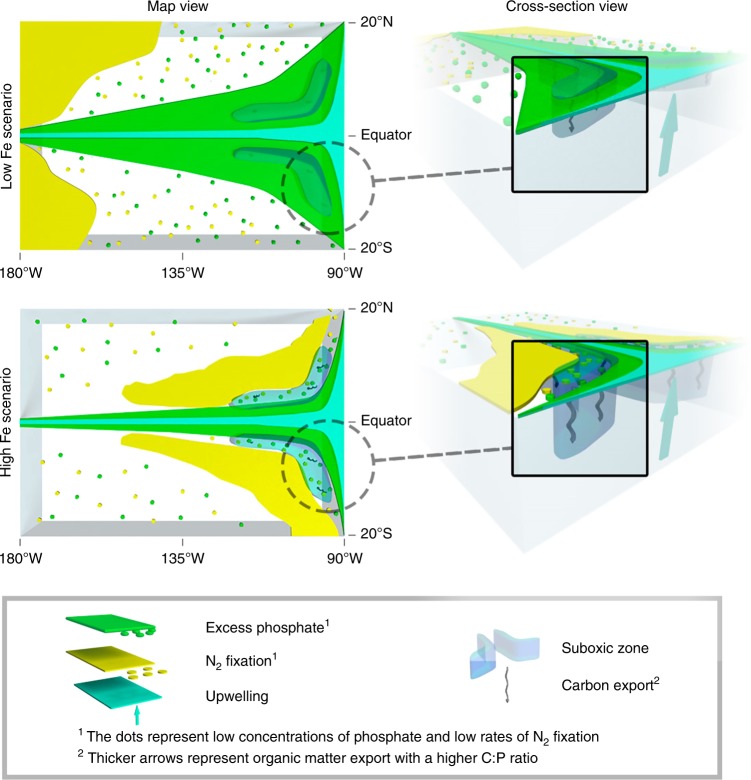
Scenarios of Fe supply to the tropical Pacific. In the low iron scenario, analogous to the modern climate, N_2_ fixation (yellow zone and dots) is concentrated in the Northwest and Southwest subtropical Pacific where aeolian dust deposition is greatest. Non-limiting PO_4_ concentrations (green zone and dots) exist within the tropics and spread laterally from the area of upwelling near the Americas and at the equator (blue zone). In the high Fe scenario, analogous to the glacial climate, N_2_ fixation couples to the upwelling zones in the east Pacific, enabling strong utilisation of PO_4_, the vertical expansion of suboxic zones (grey bubbles) and a deeper injection of carbon-enriched organic matter (downward squiggly arrows)

Our study confirms that N_2_ fixation is a key component of the global C cycle. We extend a theoretical proposal made over 20 years ago^17^ to a quantifiable mechanism of CO_2_ drawdown. The main biogeochemical feedbacks are illustrated in Fig. 5, where a coupling of N_2_ fixers to upwelling zones is the catalyst that drives CO_2_ drawdown.

The importance of N_2_ fixation for CO_2_ drawdown is relevant when assessing prior modelling work. Simulations of the glacial climate have struggled to explain the full drawdown of roughly 90 ppm^45^, unless they make manual, and therefore non-mechanistic, changes to biological functioning^38,46^. Furthermore, model studies that explore Fe fertilisation without considering variable stoichiometry and remineralisation rates^6,7^ have struggled to sequester more than 10 ppm of CO_2_. The permanent sequestration of 7–16 ppm solely via the low latitudes, therefore, represents a new and complementary pathway to explain the glacial CO_2_ drawdown. Thinking conservatively given the stratified and therefore PO_4_-limited conditions of a glacial ocean^37,42^, we propose that one third, or 10 ppm, of the 30 ppm attributed to Fe fertilisation^1^ can be explained by a closer coupling of N_2_ fixation to tropical upwelling zones.

It is important to recognise, however, that our simulations rendered eutrophic regions insensitive to Fe fertilisation. Consequently, we neglect the response of Fe-limited regions like the Southern Ocean that not only have demonstrated potential for CO_2_ drawdown^5–7,38,45^, but also influence low latitude biogeochemistry through mode and intermediate waters^47^. This work should therefore not be interpreted as a globally integrated response to Fe fertilisation. Instead, it isolates the response of the lower latitudes and offers important lessons. First, that the debated^32,48,49^ CO_2_ drawdown via the tropics is possible. Second, that this drawdown can accompany and thus complement high latitude mechanisms of CO_2_ drawdown. Third, that this drawdown requires simultaneous relief from both Fe and NO_3_ limitation^14–16^, which is plausibly achieved by stimulating N_2_ fixers with dust-borne Fe.

Our confidence in this N_2_ fixer-mediated mechanism is bolstered by our simulation of the glacial-interglacial changes in *δ*^15^N_*org*_ within the Pacific basin. However, both the drawdown of CO_2_ and the reproduction of the *δ*^15^N_*org*_ patterns in our study hinge on an acceleration of N cycling in the Eastern Tropical Pacific. By acceleration of N cycling, we mean an acceleration of the rates of N_2_ fixation and denitrification. Such an acceleration conflicts with a long-assumed deceleration of N cycling. Since Ganeshram et al.^41^, glacial records of low *δ*^15^N_*org*_ are interpreted to reflect a massive deceleration of water column denitrification, which must have exceeded a deceleration of sedimentary denitrification caused by a loss of shelf area^50^. Instead, our simulations produced an increase in sedimentary denitrification under Fe fertilisation. While both possibilities can explain the trends in Pacific *δ*^15^N_*org*_ because they both involve more sedimentary over water column denitrification, they diverge in the inferred intensity of N cycling.

New evidence questions a glacial deceleration of the N cycle in the Eastern Tropical Pacific. Recent work has revealed a vertical expansion of Pacific suboxic zones^32,51^, a feature reproduced by our Fe fertilisation simulations. While it is not well known whether sedimentary or water column denitrification is more sensitive to increases in suboxia, it seems unlikely that both would decrease as suboxic zones expanded. In fact, it seems more likely that sedimentary denitrification was stimulated as waters overlying the sediment became deoxygenated^52^ and as more organic carbon was buried within sediments^53^, while water column denitrification, which is centred within the thermocline^54^, was reduced in line with reduced rates of particle export^55^. If suboxic zones did expand vertically^32,51,52^ and local N cycling accelerated, then the coupling of N_2_ fixers to eastern upwelling zones and subsequent CO_2_ drawdown is legitimate.

The legitimacy of our proposal then requires explaining another apparent inconsistency in glacial records: how could less particle export^55^ in the tropical Pacific coincide with more C export? Our results suggest that an answer may be found in the combination of variable stoichiometry and deoxygenation. Strong PO_4_ utilisation and aeolian Fe supply enriches the C content of exported organic matter^11,56^, while deoxygenation enables a strong transfer of particles to depth^10,12,13^. If both features were present during glacial periods, then lower rates of particle export^55^ do not preclude more C export, and therefore CO_2_ drawdown.

Today, there are compelling signs that N_2_ fixation has strengthened within the Pacific since the industrial revolution^57,58^ and that suboxic zones are expanding^59,60^. Our experiments suggest that these changes are symptomatic of a stronger biological C pump, but even so, we propose that gains in N_2_ fixation remain unrealised. Evidence that N_2_ fixation is operating well below full capacity can be found in the excess PO_4_ that spreads 10–15° outwards from tropical upwelling zones^36^ (Supplementary Fig. 7b) and the spatial decoupling of N_2_ fixation from denitrification^24^. Realising the full potential of N_2_ fixation appears primarily dependent on the delivery of aeolian Fe to the surface ocean. Like the high latitudes^2,3^, we find that the strength of the lower latitude biological C pump demonstrates a strong link to the Fe cycle. However, how the oceanic Fe cycle will change in the future is uncertain^61^, and undermines our ability to predict the ocean’s role in atmospheric CO_2_ drawdown in the coming centuries.

## Methods

### Model

Model simulations were performed using the ocean component of the Commonwealth Scientific and Industrial Research Organisation (CSIRO) Mark 3L—Carbon of the Ocean, Atmosphere and Land (Mk3L-COAL) Earth system model. The ocean component is comprised of an ocean general circulation model (OGCM) described in Phipps et al.^62^ and an ocean biogeochemical model (OBGCM) described in Buchanan et al.^33^ and Buchanan et al.^34^. A more specific description of the N cycle and Fe cycle are presented in the supplement. The ocean model has a horizontal resolution of 2.8° in longitude by 1.6° in latitude, with 21 vertical levels. It is a coarse resolution, *z*-coordinate OGCM, allowing millennial timescales to be resolved.

The OBGCM is equipped with 13 prognostic tracers that can be grouped into carbon chemistry fields, oxygen fields, nutrient fields and age tracers. Carbon chemistry and air-sea gas exchange is parameterised according to the latest ocean model requirements^63^. Nitrogen isotope routines are described in Buchanan et al.^34^. The cycling of organic matter considers three forms of phytoplankton. These are a general phytoplankton group (^*G*^), N_2_ fixers (otherwise known as diazotrophs; ^*D*^) and calcifiers. The general phytoplankton group is controlled by dynamic equations for organic matter production, remineralisation and stoichiometry according to the study of Buchanan et al.^33^. These equations allow the general phytoplankton group to represent variations in the biogeochemical properties of the marine ecosystem, which has positive effects on the simulation of global ocean biogeochemistry, particularly the N cycle. Meanwhile, N_2_ fixers and calcifiers follow more static equations. N_2_ fixers have fixed nutrient limitation functions and stoichiometry based on laboratory studies, but are also remineralised according to community composition. Remineralisation of both forms of organic matter is also conserved and passed to deeper grid boxes if oxygen is not sufficient. The calcifying group, which only interacts with DIC and ALK species, produces particulate inorganic carbon at 8% of the rate at which the general phytoplankton group produces organic carbon. Its remineralisation rate is also fixed according to an *e*-folding depth-dependent decay, which transfers a large fraction of particulate inorganic carbon to the deep ocean.

### Nitrogen cycle

Nitrate is introduced to the ocean through atmospheric deposition and N_2_ fixation. Atmospheric deposition adds 11.3 Tg N to the surface ocean each year using a prescribed monthly climatology^64^.

The addition of NO_3_ by N_2_ fixation is calculated by considering marine N_2_ fixers as a unique group of phytoplankton. N_2_ fixers consume PO_4_ and Fe at the surface ocean, and release PO_4_, Fe and NO_3_ at depth during remineralisation. The stoichiometry of N_2_ fixers is static, with a C:N:P:Fe ratio of 331:50:1:0.00064 according to physiological studies^39,65,66^. With this stoichiometry, we apply O_*rem*_:P and N_*rem*_:P requirements of 431 and 294.8, respectively, using the equations of Paulmier et al.^67^.

The export of phosphorus by N_2_ fixers $(P_{exp}^D)$ is calculated using a maximum growth rate μ^*D*^(*T*) that is temperature dependent^68^, limitation terms dependent on the availability of PO_4_, NO_3_ and Fe, and minimum thresholds to account for cold water N_2_ fixation^69^. These terms are applied against an export:production ratio $(S_{E:P}^D)$ in units of mmol P m^−3^ day^−1^. $P_{exp}^D$ is calculated via:

1 $$P_{exp}^D = S_{E:P}^D \cdot \mu ^D(T) \cdot {\mathrm{max}}(0.01,{\mathrm{{min}}}(PO_{4_{lim}^D},NO_{3_{lim}^D},Fe_{lim}^D)) \cdot (1 - ico)$$

where,

$$\mu ^D(T) = {\mathrm{max}}(0.01, - 0.0042T^2 + 0.2253T - 2.7819)$$

$$PO_{4_{lim}^D} = \frac{{PO_4}}{{PO_4 + K_{PO_4}^D}}$$

$$NO_{3_{lim}^D} = e^{ - NO_3}$$

$$Fe_{lim}^D = {\mathrm{max}}(0.0,{\kern 1pt} {\mathrm{{tanh}}}(2Fe - K_{Fe}^D))$$

The Fe half saturation coefficient ($K_{Fe}^D$) was kept at 0.3 μmol m^−3^, 3× that of other phytoplankton, unless otherwise clearly defined as another value in our discussion of the results below. The PO_4_ half saturation coefficient ($K_{PO_4}^D$) was 10^−10^ unless otherwise clearly defined as another value to emulate N_2_ fixers efficient utilisation of P^25,26^. Light was also not considered as a limiting factor. A dependency on light was omitted because of the strong correlation between incident radiation and sea surface temperature^70^ and its negligible effect on N_2_ fixation in the Atlantic Ocean^71^. Finally, the fractional area coverage of sea ice (*ico*) is included to ensure that no cool-water N_2_ fixation^69^ occurs under ice. The remineralisation of N_2_ fixer export occurs at the same rate as other labile organic matter produced by the general phytoplankton group.

Two processes remove NO_3_ from the ocean model: water column and sedimentary denitrification. Water column denitrification occurs when O_2_ concentrations are less than a particular threshold $(R_{lim}^{O_2})$, which is set at 7.5 mmol O_2_ m^−3^. We calculate the fraction of organic matter (*P*_*org*_) that is remineralised by water column denitrification via:

2 $$f_{den} = \left( {1 - e^{ - 0.5 \cdot R_{lim}^{O_2}} + e^{{\mathrm{{O}}}_2 - 0.5 \cdot R_{lim}^{{\mathrm{{O}}}_2}}} \right)^{ - 1}$$

and then apply the appropriate stoichiometric requirements of NO_3_ to this fraction of *P*_*org*_:

3 $$\Delta NO_3(WC_{den}) = f_{den} \cdot P_{org} \cdot {\mathrm{{N}}}_{rem}:{\mathrm{{P}}}$$

Following this, the strength of water column denitrification is reduced if the ambient concentration of NO_3_ is deemed to be limiting. Water column denitrification depletes NO_3_ towards concentrations between 15 and 40 mmol m^−3^ in modern suboxic zones^36^. Without this additional constraint, here defined as *r*_*den*_, NO_3_ concentrations quickly go to zero in simulated suboxic zones. We calculate *r*_*den*_ by prescribing a lower limit at which NO_3_ can no longer be consumed $(R_{lim}^{NO_3})$, which was set to 30 mmol NO_3_ m^−3^:

4 $$r_{WC_{den}} = 0.5 + 0.5 \cdot {\mathrm{{tanh}}}(0.25 \cdot {\mathrm{{NO}}}_3 - 0.25 \cdot R_{lim}^{{\mathrm{{NO}}}_3} - 2.5)$$

5 $${\mathrm{if}}\quad r_{WC_{den}} < {\hskip 3pt} f_{den},\quad {\mathrm{then}}\quad f_{den} = r_{WC_{den}}$$

Sedimentary denitrification was calculated using the paramaterisation of Bohlen et al.^72^, where the removal of NO_3_ is dependent on the rain rate of organic carbon to the sediments (*C*_*org*_) and the ambient concentrations of O_2_ and NO_3_.

6 $$\Delta {\mathrm{{NO}}}_3(S_{den}) = \left( {\alpha + \beta \cdot 0.98^{({\mathrm{{O}}}_2 - {\mathrm{{NO}}}_3)}} \right) \cdot C_{org}$$

The *α* term was 0.08, while the *β* term was halved compared the original value of Bohlen et al.^72^ to *β* = 0.1 in an attempt to increase the deep NO_3_ inventory. The availability of NO_3_ for sedimentary denitrification was accounted for according to the equation:

7 $$r_{S_{den}} = 0.5 + 0.5 \cdot {\mathrm{tanh}}(10 \cdot {\mathrm{{NO}}}_3 - 5)$$

Thus, sedimentary denitrification was relaxed towards zero as NO_3_ concentrations became low.

If NO_3_ was limiting, the remaining organic matter was remineralised using O_2_, so long as the environment was sufficiently oxygenated. The availability of oxygen in the sediments was estimated to be two-thirds of the overlying bottom water concentration, based on observations of transport across the diffusive boundary layer by Gundersen and Jorgensen^73^. Furthermore, an additional limitation was set for sediments underlying hypoxic waters (O_2_ < 40 mmol m^−3^), where aerobic remineralisation was diminished towards zero according to the hyperbolic tangent function:

8 $$r_{S_{rem}} = 0.5 + 0.5 \cdot {\mathrm{tanh}}(0.2 \cdot {\mathrm{{O}}}_2 - 5)$$

If both NO_3_ and O_2_ were limiting, the remaining organic matter was assumed to be remineralised via sulfate reduction.

### Subgrid-scale bathymetry

A large amount of sedimentary remineralisation was not included using these parameterisations because the coarse resolution OGCM enables it to resolve only the largest continental shelves. Many small areas of raised bathymetry in pelagic environments were also unresolved. To address this insufficiency, we coupled a sub-grid scale bathymetry to the course resolution OGCM following the methodology of Somes et al.^74^ and using the ETOPO5 $\frac{1}{{12}}^{th}$ of a degree dataset. For each latitude by longitude grid point, we calculated the fraction of area that would be represented by shallower levels in the OGCM if this finer resolution bathymetry were used. At each depth level above the OGCM’s deepest level, the fractional area represented by sediments on the sub-grid scale bathymetry was used to remineralise all forms of organic matter via the sedimentary processes defined above.

### Iron cycle

Our simulated Fe cycle involves a prescribed external source via the aeolian deposition of dust^35^, and an internal control in water masses in contact with the ocean floor. The internal control relaxes Fe concentrations to a set concentration given in the control file, which is set to 0.6 μmol m^−3^ over a period of 1 year. The iron cycle, therefore, considers an atmospheric source, internal cycling via organic matter, and deep ocean sources and sinks via the sediments.

### Simulations

All experiments were simulated for 10,000 years to achieve steady-state solutions of major biogeochemical tracers. Unless clearly defined otherwise, all experiments were run under preindustrial conditions, Mk3L^*mild*^, driven by monthly climatologies of surface conditions over an annual cycle. Surface climatologies required to force the OGCM and OBGCM under Mk3L^*mild*^ conditions were generated by a 10,000 year pre-industrial (PI) control run of the flux corrected CSIRO Mk3L v1.2 climate system model in fully coupled mode^62^.

We forced the OGCM with three sets of additional boundary conditions to generate cold, cool and warm ocean states in addition to Mk3L^*mild*^. The glacial ocean state (Mk3L^*cold*^) was generated by forcing the CSIRO Mk3L climate system model with glacial conditions as simulated in Buchanan et al.^38^. Warm and mild conditions of GFDL^*warm*^ and HadGEM^*cool*^, respectively, were provided by the pre-industrial control runs of the GFDL-ESM2G and HadGEM2-CC climate system models from the Climate Model Inter-comparison Project phase 5 (CMIP5) multi-model ensemble^75^. More thorough physical analyses of these ocean states are contained in Buchanan et al.^38^ and Buchanan et al.^33^.

Iron deposition experiments that varied Fe supply to the surface ocean involved altering the field of Mahowald et al.^35^ with constant factors to achieve 25, 50, 75, 80, 90, 100, 125, 150, 200, 300, and 400% of the modern flux. Higher fluxes representative of the glacial field were undertaken using the dust deposition fields of Lambert et al.^5^ assuming 3.5% Fe content and 0.4 and 2% solubility, respectively, to achieve 500 and 2500% of the modern Fe supply rate (Supplementary Fig. S2). The glacial dust deposition rate referred to in the main text is the 500% version of the Lambert et al.^5^ field (Supplementary Fig. 2). These rates of Fe deposition were applied to the Mk3L^*mild*^ state and discussed in “A central role for N_2_ fixers”, while a subset of these Fe deposition experiments, as well as variations in the Fe half-saturation constant for N_2_ fixers (see Supplementary description of the N cycle), were undertaken in with multiple physical states discussed in “Quantifying CO_2_ drawdown”.

For those experiments with a freely evolving atmospheric CO_2_ concentration (within section “Quantifying CO_2_ drawdown”), we initialised each with the near-equilibrium solution produced by holding atmospheric *p*CO_2_ at 280 ppm and with the modern Fe deposition (stars in Fig. 2), such that experiments with modern Fe deposition maintained atmospheric *p*CO_2_ near to 280 ppm. Altering Fe deposition then caused changes in air-sea CO_2_ exchange that altered the atmospheric and oceanic C reservoirs. The atmospheric C reservoir was calculated assuming a constant atmospheric weight of 5.1 × 10^21^ g and a mean molecular weight of air of 28.97 g mol^−1^.

All experiments involved a relaxation of deep ocean Fe to values of 0.6 μmol m^−3^ over a period of 365 days. Areas of connection between the deep and surface ocean, such as the high latitudes and deep upwelling zones, were therefore either non-Fe limited or almost non-Fe limited. This parameterisation rendered the high latitudes insensitive to greater Fe supply, while stratified lower latitudes were sensitive to Fe supply but NO_3_-limited.

### *δ*^15^N_*org*_ records

Glacial minus interglacial values of *δ*^15^N_*org*_ records were calculated by averaging values during the Last Glacial Maximum, defined as between 20 and 26 kya, and the Late Holocene, defined as between 0–5 kya. The early Holocene was ignored due to transient changes in the *δ*^15^N records since the deglaciation. The global compilation of *δ*^15^N_*org*_ was composed of bulk sediment and diatom- and foraminifera-bound measurements, and is available in the supplementary material. A slight correction to simulated *δ*^15^N_*org*_ was applied to correct for diagenetic effects that increase with depth in the water column. The addition of 0.9 per 1000 metres to the raw, simulated *δ*^15^N_*org*_ values was applied and substantially improves comparisons between simulated and coretop values^34^.

## Supplementary information

Supplementary Information

Peer Review File

Description of Additional Supplementary Files

Supplementary Data 1

Source Data

## Data Availability

The model output data that support the findings of this study are available for download from Australia’s National Computing Infrastructure (NCI) at https://researchdata.ands.org.au/marine-nitrogen-fixers-output-v10/1385710 with the identifier 10.25914/5d730c40c2729. Source data underlying Figs. 2 and 3 are provided in the Supplementary information as a source data file. Glacial-interglacial differences in *δ*^15^N_*org*_ are held in Supplementary Data 1. Code for making Figs. 1–4 is freely available at https://github.com/pearseb/Marine-nitrogen-fixers-paper-python-code.

## Code availability

The source code for CSIRO Mk3L-COAL is shared via a repository located at http://svn.tpac.org.au/repos/CSIRO_Mk3L/branches/CSIRO_Mk3L-COAL/. Access to the repository may be obtained by following the instructions at https://www.tpac.org.au/csiro-mk3l-access-request/. Access to the source code is subject to a bespoke license that does not permit commercial usage, but is otherwise unrestricted. An “out-of-the-box” run directory is also available for download with all files required to run the model in the configuration used in this study, although users will need to modify the *runscript* according to their computing infrastructure. Any queries may be directed to the lead author.
