# Supplementary material for: Marine nitrogen fixers mediate a low latitude pathway for atmospheric CO2 drawdown
Source: Nat Commun. 2019 Oct 10;10:4611. doi: 10.1038/s41467-019-12549-z (PMC6787065; doi:10.1038/s41467-019-12549-z)
Supplement: Supplementary file 5 — Source Data [file 41467_2019_12549_MOESM5_ESM.pdf]

Supplementary Information to “Marine nitrogen fixers mediate a low latitude pathway for atmospheric CO_2 drawdown”

Buchanan et al., Nature Communications (2019)

Supplementary Note 1

Global temperature, salinity and δ^{14}C fields produced by the CSIRO Mk3L control (Mk3L^{mild}) were in good agreement with observations (Supplementary Figure 3; Supplementary Table 1). Global and surface correlations of temperature were excellent at ≥ 0.98, as is expected of boundary conditions produced by a flux corrected coupled model. The fit of global and surface salinity was worse at ≥ 0.87, with waters deeper than 2,000 metres being too fresh by 0.27 psu. This reflects an inability of the OGCM to achieve peak wintertime densities in high latitudes, which is partly caused by enforced relaxation to the prescribed surface climatologies[1].

Overturning in each major basin (Supplementary Figure 4) involved an Atlantic dominated by an upper cell of North Atlantic origin but with dense southern source waters beneath 2,500 metres, a Pacific Ocean dominated by a lower cell of southern origin, and an Indian Ocean with sluggish deep circulation. The formation rates of key water masses, responsible for producing this circulation, mostly fell within the range provided by estimates from the literature (Supplementary Table 1). The only exception was the overturning of intermediate waters produced in the North Pacific, which was too strong, and the Southern Ocean, which was too weak. The degree to which each basin was affected by the lower cell, and thus waters of southern origin, was a good estimate for the residence time of that basin. The Pacific ocean contained the oldest waters, followed by the mid-depth waters in the Indian Ocean, as evident from the δ^{14}C distribution.

However, the age of deep waters in all ocean basins was slightly too old when comparing simulated δ^{14}C to the observations[2], particularly in the North Pacific and North Atlantic. This may

partly explain the existence of anoxic zones that were too intense and too deep. The volume of anoxic water ($O_2 < 1$ mmol m^{-3}) as a percentage of the ocean was 1.9 %, much greater than the 0.3 % of observations[3]. Large volumes of anoxic water are an unfortunate feature of coarse resolution OGCMs with poorly resolved equatorial undercurrents[4]. The oxygen minimum zone of the North Indian Ocean was also positioned in the Bay of Bengal, rather than the Arabian Sea, which is another common feature of coarse resolution OGCMs[5,6]. Despite these obvious inconsistencies, a mean oxygen concentration of about 185 mmol m^{-3} and hypoxic water ($O_2 < 50$ mmol m^{-3}) volumes varying between 4.6 and 5.5 % were in better agreement with observations of 176 mmol m^{-3} and 4.4 %[7].

For a more detailed description and evaluation of the ocean dynamics see Phipps *et al.*[1,8,9].

Supplementary Note 2

A global increase in NO_3 of 4.3 mmol m^{-3} was enforced by tuning down the α constant in equation 6 (see methods in main article) from 0.08 to 0.0008. The increase in NO_3 was most apparent in the high latitudes, where waters were rapidly mixed between surface and subsurface layers (Supplementary Figure 6). However, small increases in NO_3 in the oligotrophic regions increased PO_4 and Fe utilisation, increasing the community C:P ratio and elevating C export. However, this increase was offset by a decrease in N_2 fixer C export (Supplementary Figure 6), which stabilised global C export. As a result, little change in the C inventory occurred.

Supplementary Table 1: Estimates of key physical diagnostics for Mk3L^{mild}. Temperature (T), salinity (S) and oxygen (O_2) are global averages. Other metrics are important water mass transports in Sverdrups (10^6 m^{-3} s^{-1}) compared with estimates from the literature[10–17]. The formation rate of Antarctic Bottom Water (AABW) was calculated as the minimum global overturning circulation south of 60°S between the surface and 2,000 metres. The formation rate of North Atlantic Deep Water (NADW) was calculated as the maximum overturning rate in the North Atlantic Ocean north of 30°N and between the surface and 2,000 metres. The formation rate of North Pacific Intermediate Water (NPIW) was calculated as the maximum overturning rate in the North Pacific Ocean north of 30°N and between the surface and 1,000 metres. The transport of water in the Antarctic Circumpolar Current (ACC) was calculated as the time-averaged maximum barotropic transport through Drake Passage. The subduction / upwelling of Southern Source Intermediate Water (SSIW), composed of both Antarctic Intermediate Water and Subantarctic Mode Water, was calculated by determining the locations of isopycnal outcropping and the transports across the mixed layer at these points according to Appendix B of Buchanan et al.[18]. All values represent annual averages of the monthly metrics.

	T (°C)	S (psu)	O_2 (mmol m^{-3})	AABW^a Sv	NADW^b Sv	NPIW^c Sv	SSIW^d Sv	ACC^e Sv
Obs	4.0	34.72	176	12.5 ± 4	18 ± 5	2.3 ± 0.1	11.9 / 6.1	134-173
Mk3L$_{PI}^{mild}$	4.0	34.50	185	11.1	18.4	11.1	6.5 / 5.2	166

^{a}Estimates from Lumpkin *et al.*[15] (contains Orsi *et al.*[12])

^{b}Estimates from Talley *et al.*[13] (contains others[14,15])

^{c}Estimates from Lumpkin *et al.*[15] and Talley *et al.*[13]

^{d}Combined subduction / upwelling rate of AAIW and SAMW from Iudicone *et al.*[17]

^{e}Range provided by Cunningham *et al.*[19] and Donohue *et al.*[20]

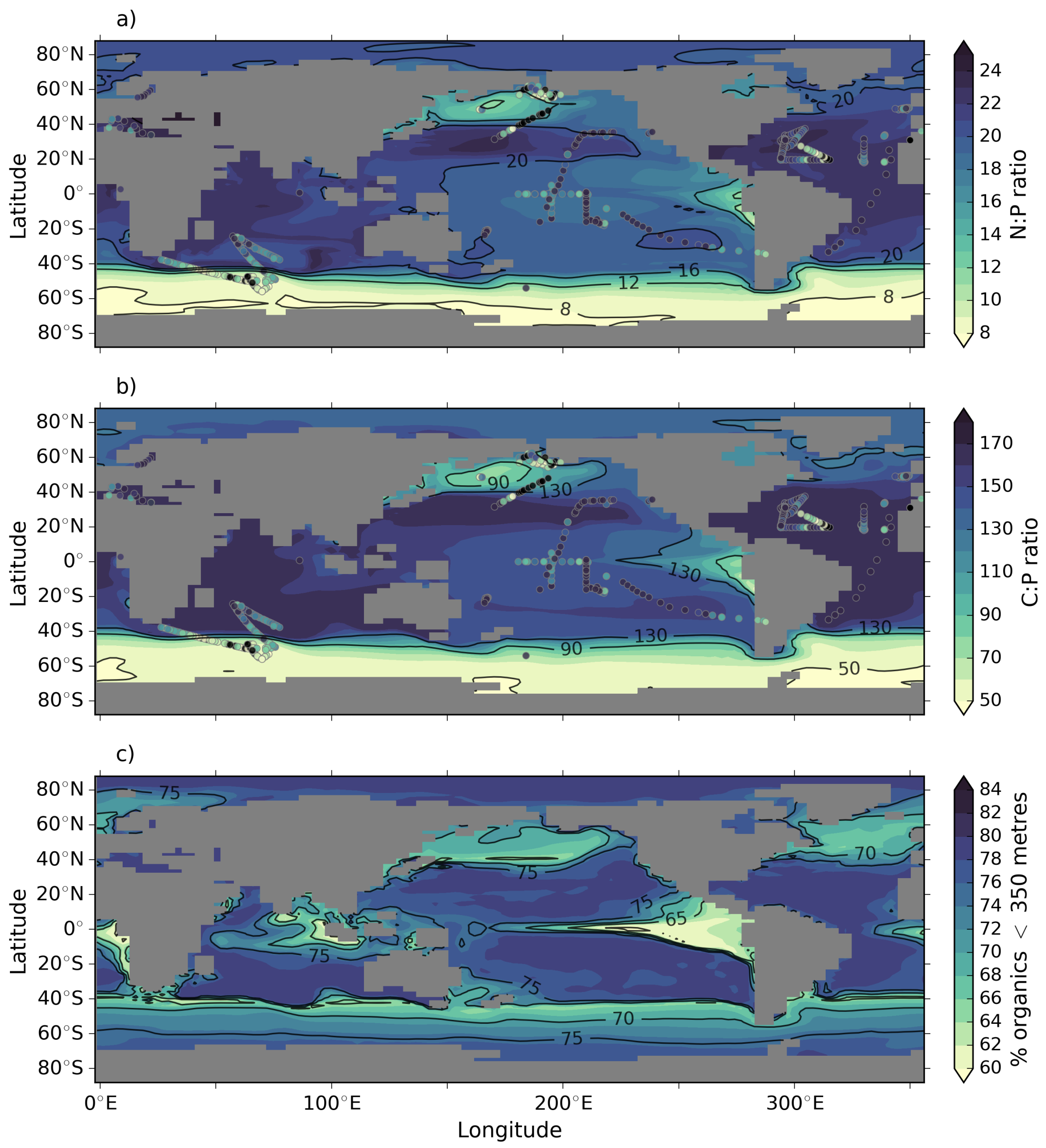

Supplementary Figure 1: **Demonstration of variable ecosystem properties.** Annual average (a) nitrogen to phosphorus ratios and (b) carbon to phosphorus ratios of the non-N_2 fixer community. The stoichiometry and transfer efficiency of marine organic matter were dynamically calculated at each timestep using the equations applied in the study of Buchanan *et al.*[18]. These dynamic equations account for the wide, regional variations in C:N:P ratios and remineralisation rates known of the global ocean. The data compilation of stoichiometric observations[21] is overlain on both plots. Panel (c) depicts the % of organic matter that is remineralised in the upper 350 metres.

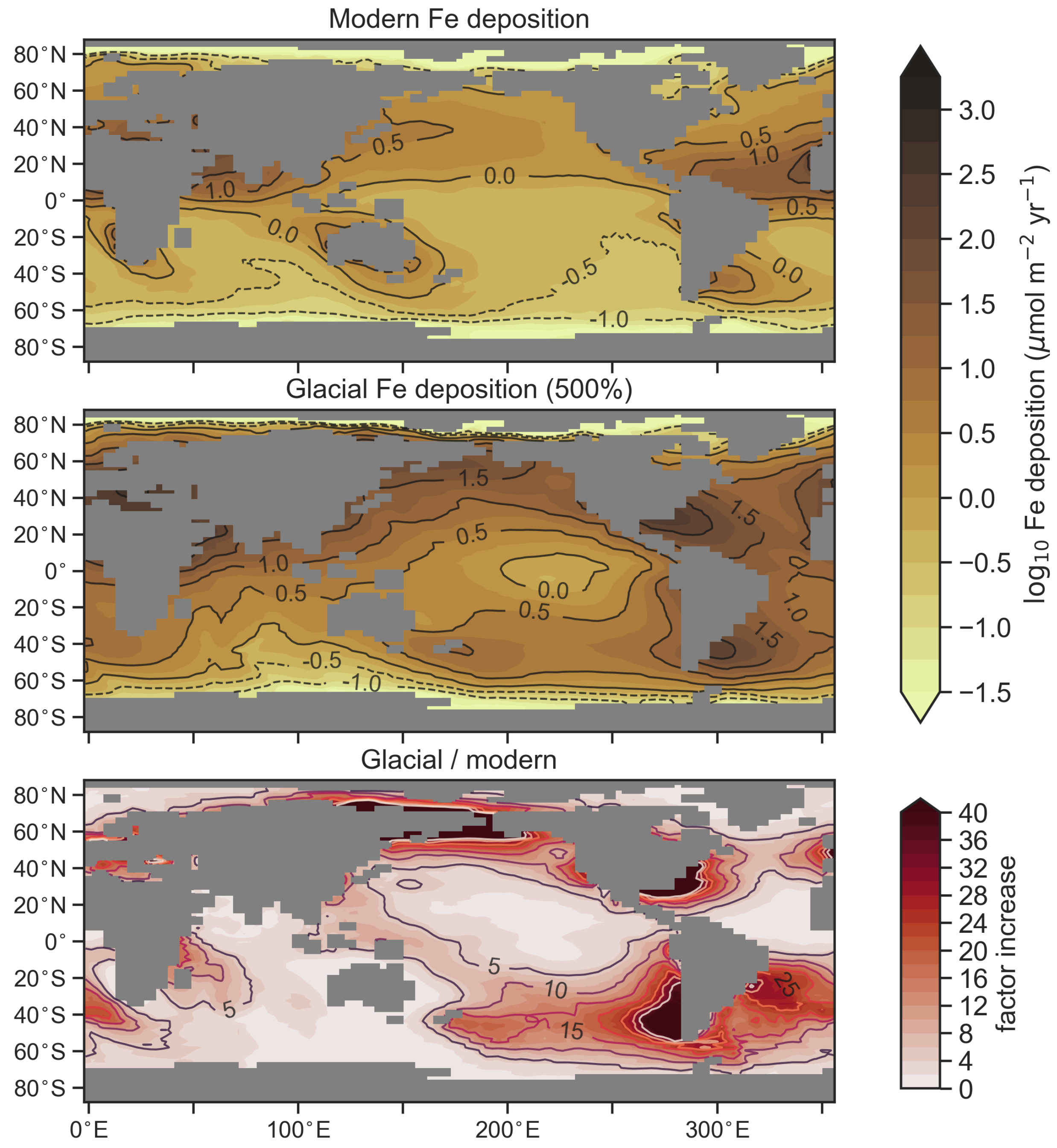

Supplementary Figure 2: **Aeolian deposition fields of Fe in dust.** Modern (top) and glacial (centre) scenarios are show, as well as the factor increase in Fe deposition under the glacial scenario (bottom). The modern field was provided by Mahowald *et al.*[22]. The glacial field was provided by the climatology of dust of Lambert *et al.*[23], assuming 3.5 % iron content and 0.4 % solubility.

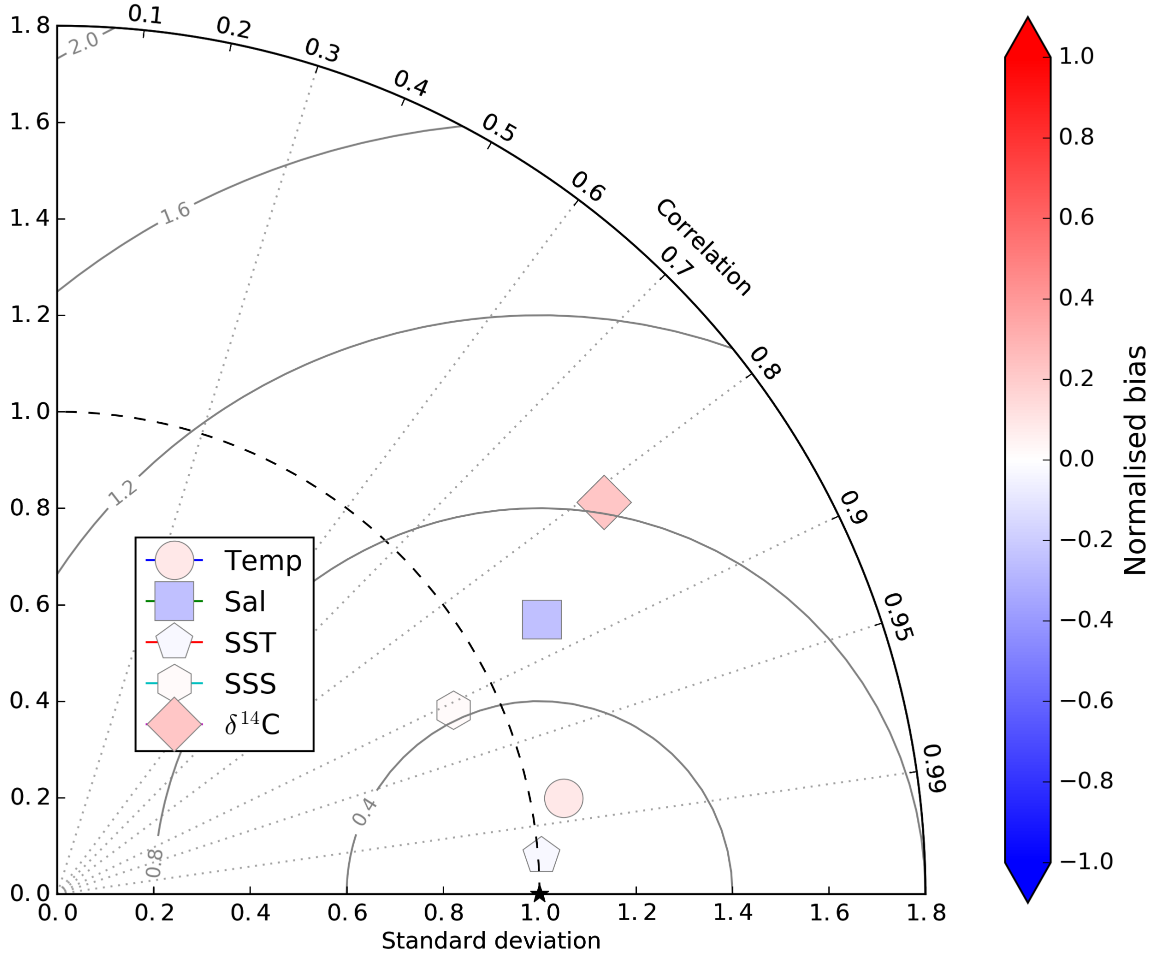

Supplementary Figure 3: **Measures of model-data fit of Mk3L^{mild}.** Taylor diagram[24] showing univariate statistical measures of fit between simulated and observed (the star) fields of temperature, salinity, surface temperature, surface salinity and δ^{14}C. Measures of fit are the correlation coefficient (angle), normalised root mean square error (solid curved lines), normalised standard deviation (distance from dashed curve), and normalised bias (colour).

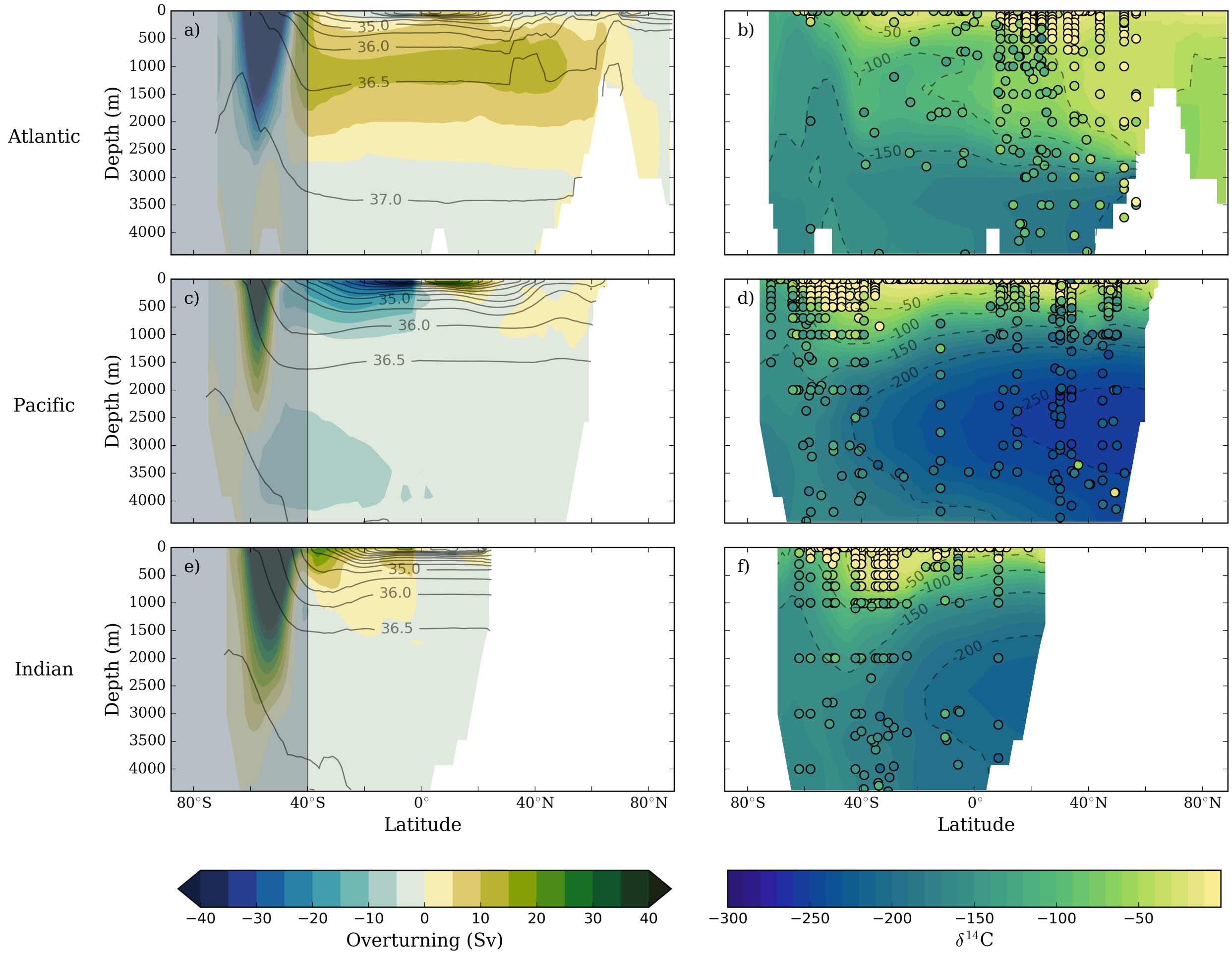

Supplementary Figure 4: **Visualising the circulation of Mk3L^{mild}.** Simulated overturning in Sverdrups (1 Sv = 10^6 m^3 s^{-1}) within the (a) Atlantic, (c) Pacific and (e) Indian Oceans. Zonal mean δ^{14}C measured in ‰ for (b) the Atlantic, (d) Pacific, and (f) Indian Oceans. Overturning south of 40°S is excluded because water can exit to the east or west, and the streamfunction does not account for these losses. The overturning streamfunction is overlain by zonally averaged contours of potential density (kg m^{-3} minus 1,000) referenced to 2,000 metres. The simulated δ^{14}C distribution of the ocean (contours) is overlain with the compilation of historical measurements by Graven *et al.*[2].

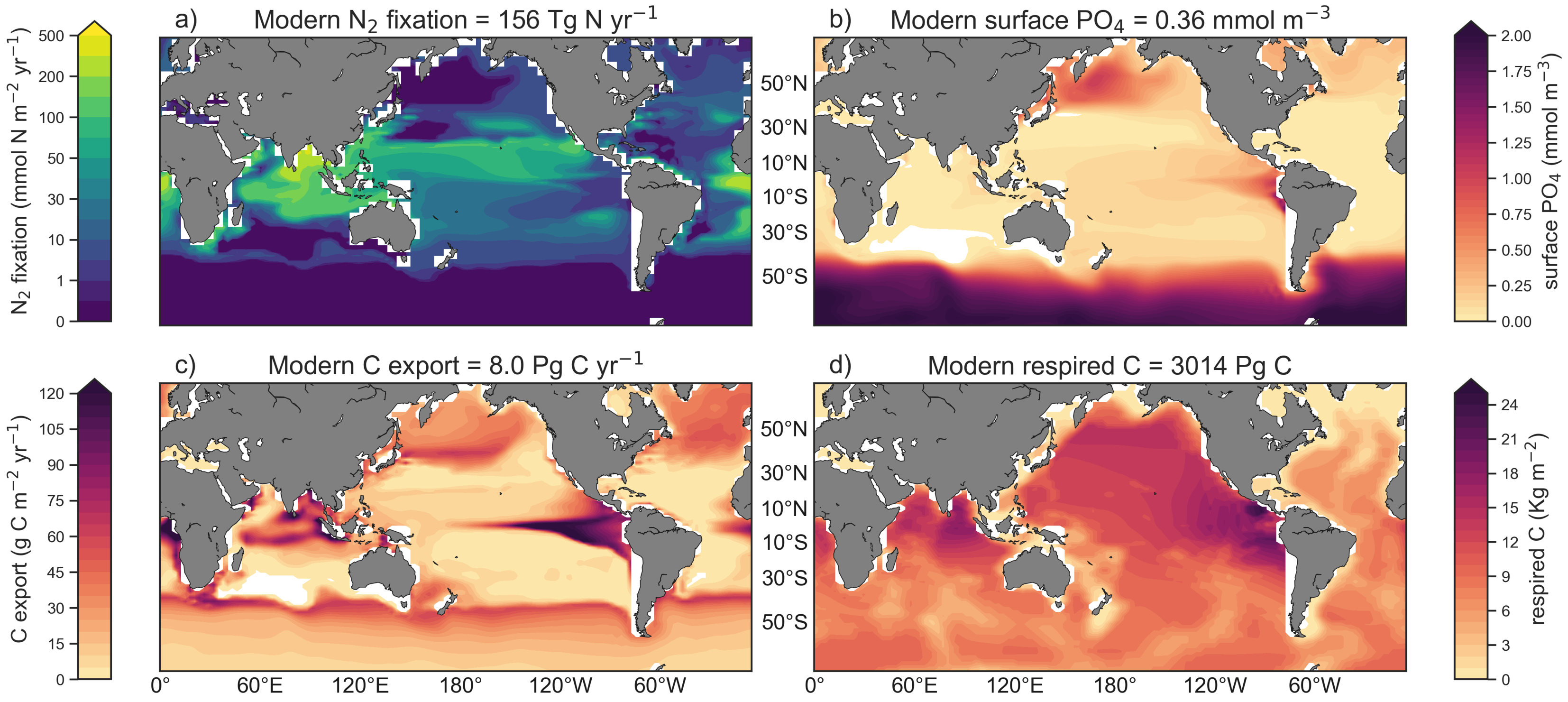

Supplementary Figure 5: **Biogeochemical properties of Mk3l^{mild} under modern Fe supply.** (a) N_2 fixation rate, (b) surface PO_4 concentration, (c) C export rate, and (d) respired C content. All fields are annual averages.

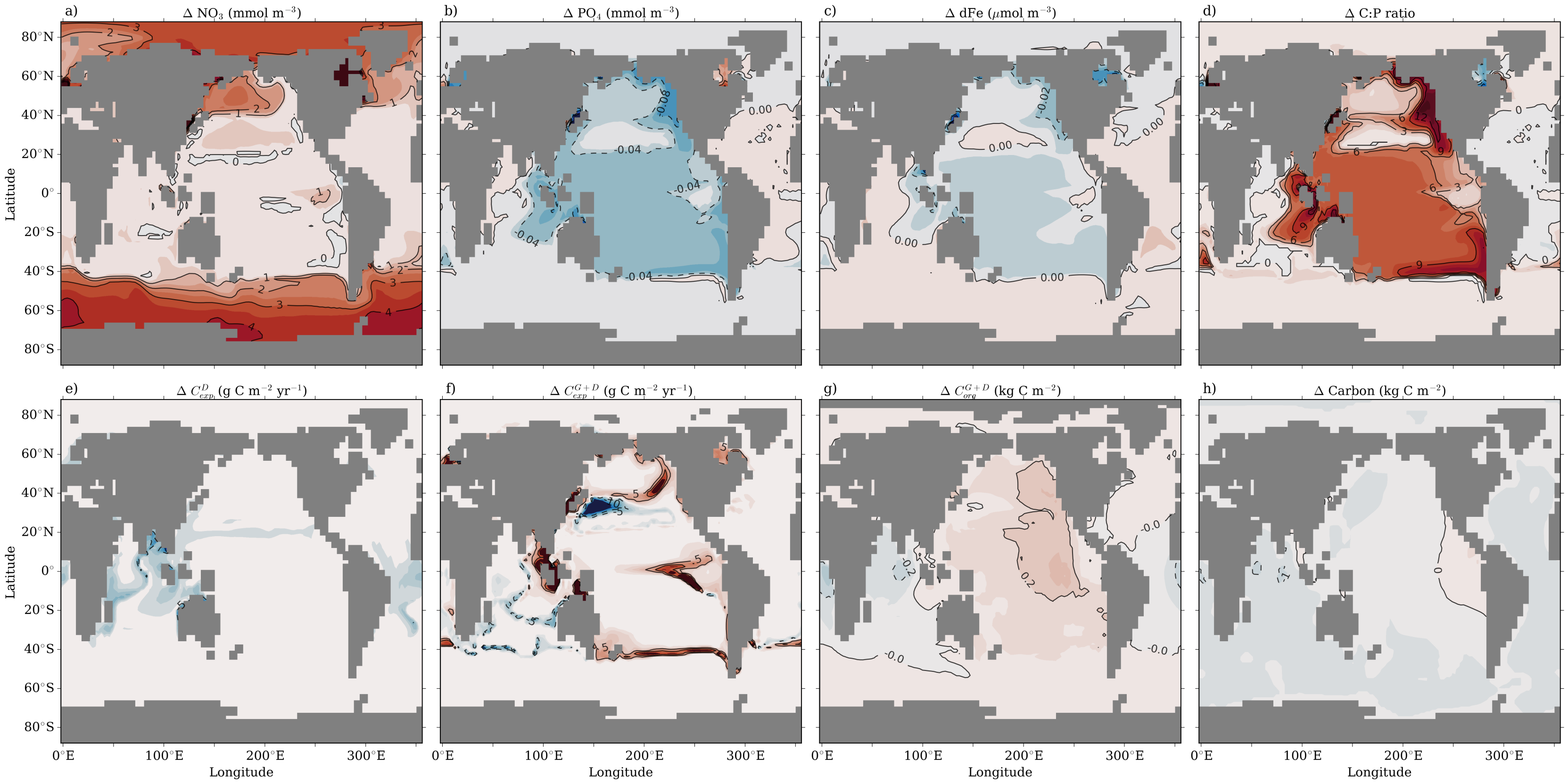

Supplementary Figure 6: **Biogeochemical response to an increase in the NO_3 inventory.** Change in surface features of the marine ecosystem and depth-integrated carbon content are shown with an increase in average NO_3 concentration of 4.3 mmol m^{-3}. (a) surface nitrate, (b) surface phosphate, (c) surface dissolved iron, (d) C:P stoichiometry of organic matter (C^{G}_{exp}), (e) carbon export by N_2 fixers (C^{D}_{exp}), (f) total carbon export of the marine ecosystem (C^{G+D}_{exp}), (g) depth-integrated respired carbon (C^{G+D}_{org}), and (h) depth-integrated carbon content.

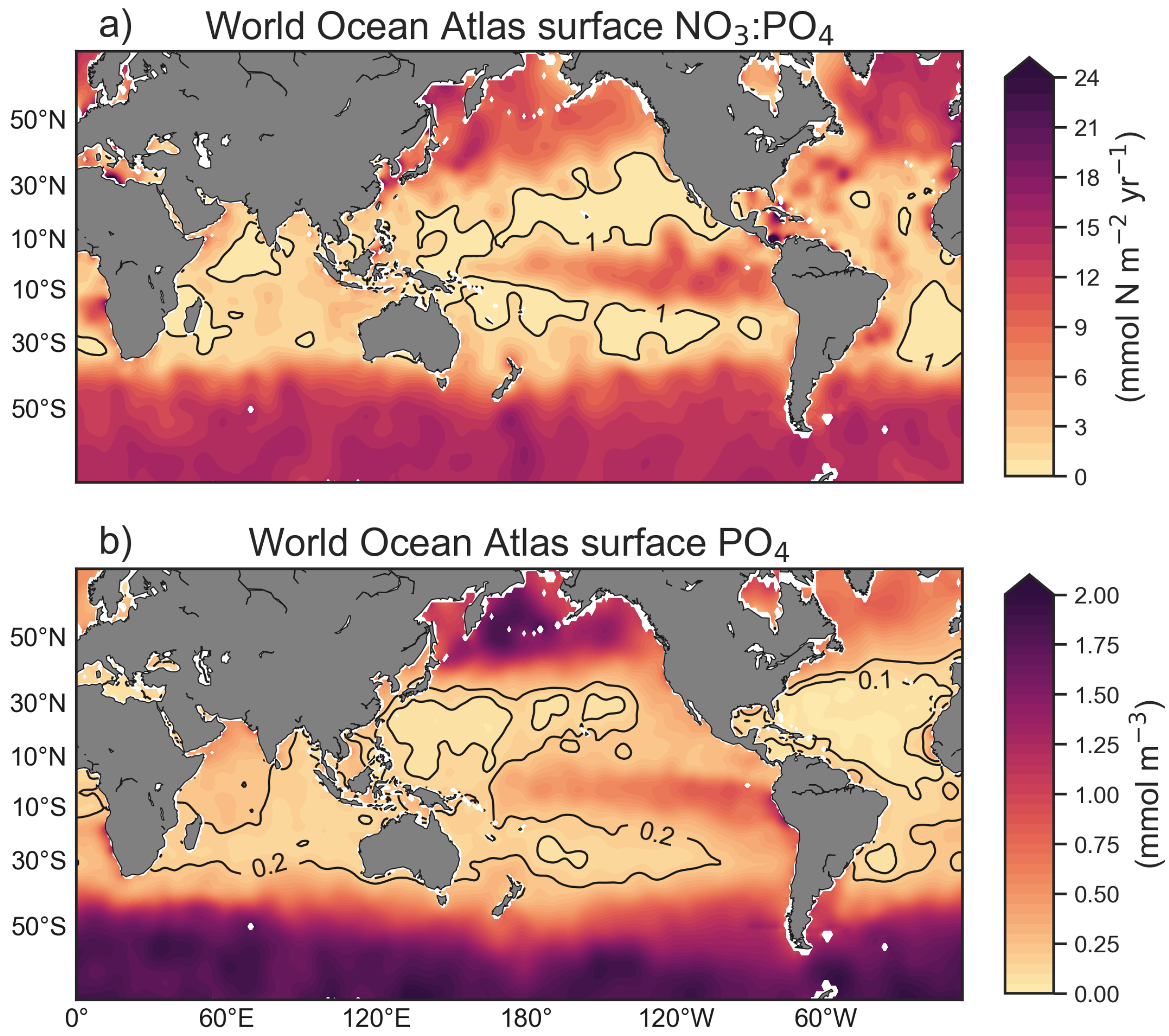

Supplementary Figure 7: **Concentrations of surface nutrients according to the World Ocean Atlas[25].** (a) NO_3:PO_4 concentration, and (b) surface PO_4 concentration. All fields are annual averages. Contours illustrate thresholds discussed in the text.

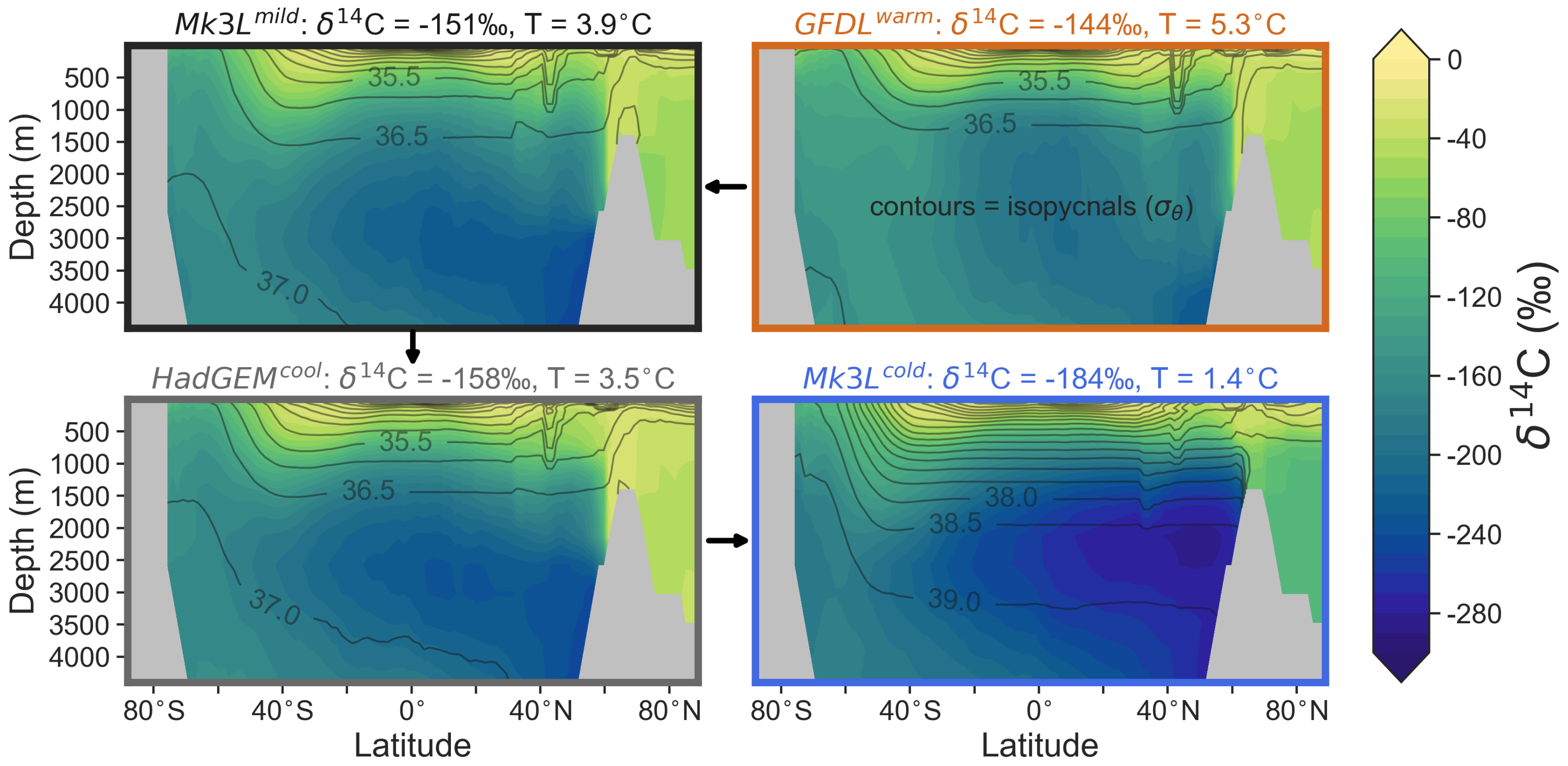

Supplementary Figure 8: **Comparing the four ocean circulation states.** Radiocarbon (shading) and potential density referenced to 2,000 metres (contours) of the four circulation states. Follow the arrows to go from a warm, well-mixed, well ventilated ocean to a cold, stratified, poorly ventilated ocean. Global mean values of radiocarbon and temperature are given at the top of each figure.

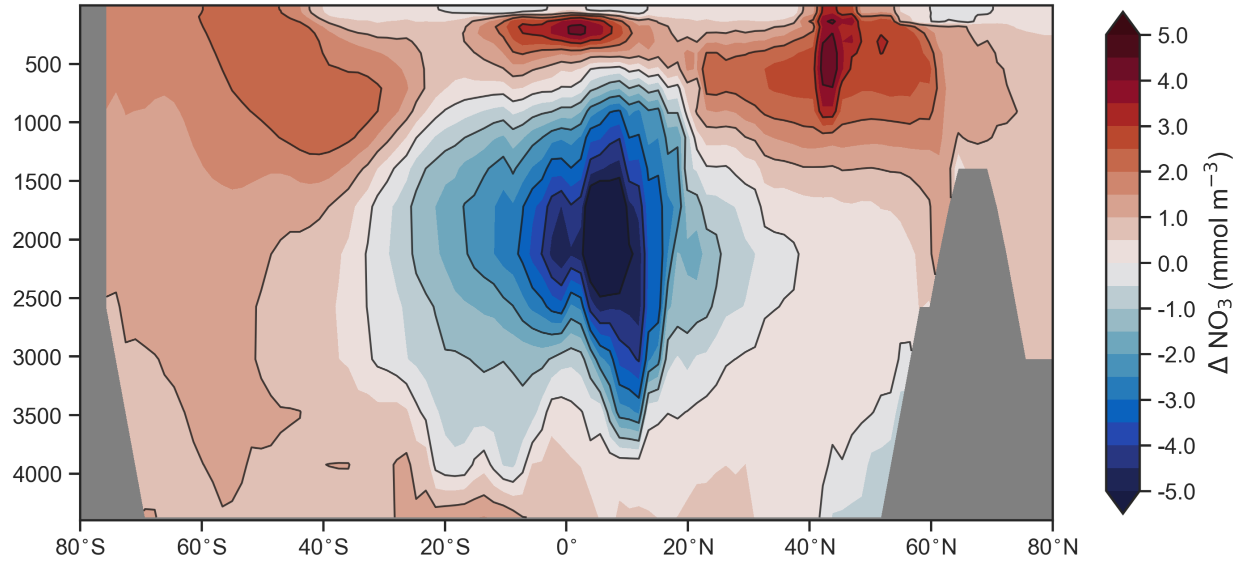

Supplementary Figure 9: **Global response of NO_3 to the presence of N_2 fixers.** Change in the annual, zonal mean of NO_3 is shown due to the introduction of N_2 fixers under the Mk3L^{mild} ocean state.

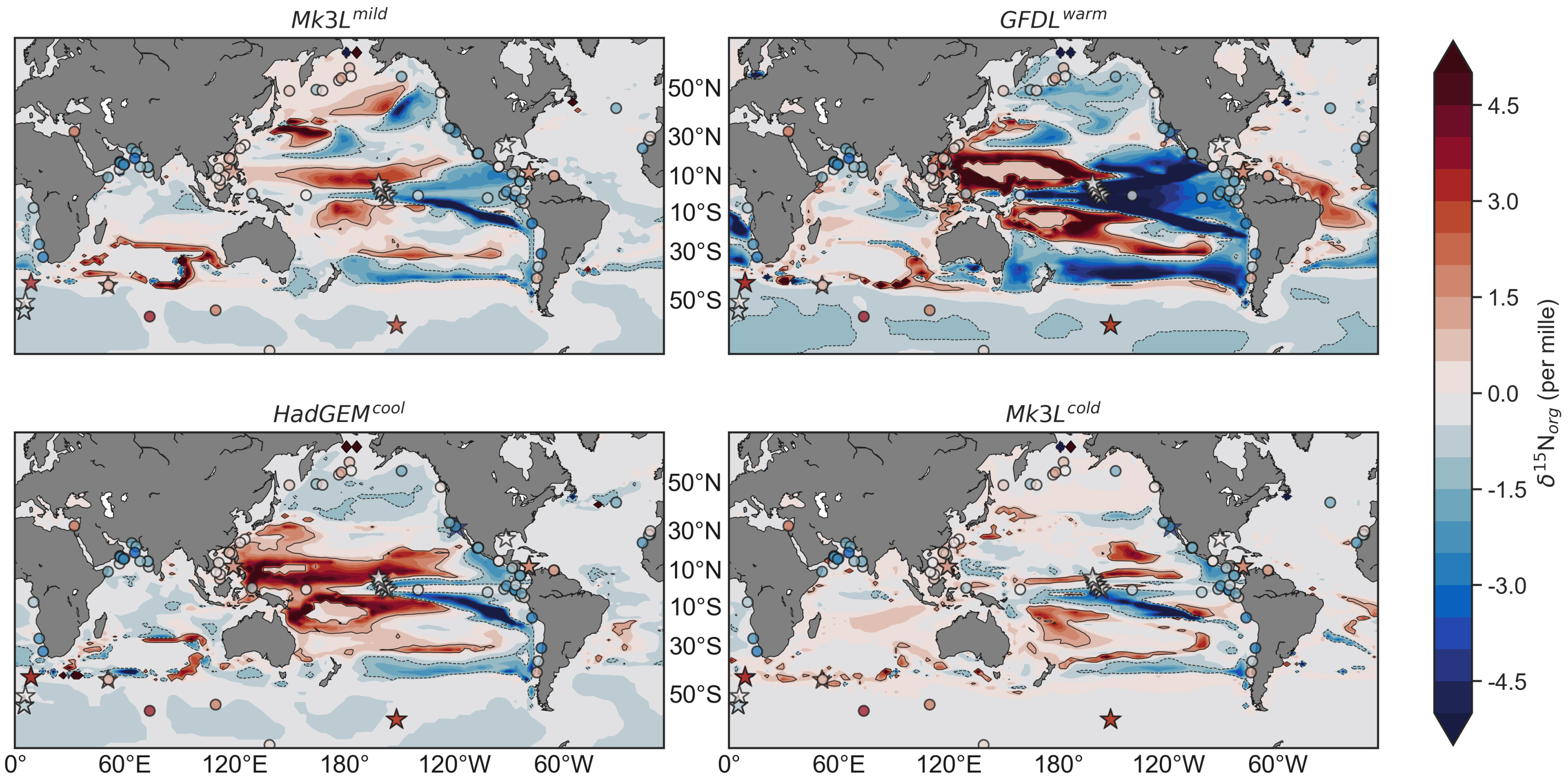

Supplementary Figure 10: **Simulated changes in δ^{15}N of organic matter to glacial Fe supply.** Aeolian Fe supply was increased from the modern to a glacial rate in all four ocean states. Circles mark locations of sediment cores where bulk organic matter was analysed for δ^{15}N, while stars mark locations where either foraminifera- or diatom-bound δ^{15}N was measured (Supplementary Data 1). The colour of the markers is an estimate of the glacial-interglacial difference.

Supplementary References

1. Phipps, S. J. *et al.* The CSIRO Mk3L climate system model version 1.0 - Part 1: Description and evaluation. *Geoscientific Model Development* **4**, 483–509 (2011).

2. Graven, H., Kozyr, A. & Key, R. Historical observations of oceanic radiocarbon conducted prior to geosecs (2012).

3. Bianchi, D., Dunne, J. P., Sarmiento, J. L. & Galbraith, E. D. Data-based estimates of suboxia, denitrification, and N 2 O production in the ocean and their sensitivities to dissolved O 2. *Global Biogeochemical Cycles* **26**, n/a–n/a (2012).

4. Brandt, P. *et al.* Oxygen tongues and zonal currents in the equatorial Atlantic. *Journal of Geophysical Research: Oceans* **113**, 1–15 (2008).

5. Moore, J. K. & Doney, S. C. Iron availability limits the ocean nitrogen inventory stabilizing feedbacks between marine denitrification and nitrogen fixation. *Global Biogeochemical Cycles* **21**, 1–12 (2007).

6. Oschlies, A., Schulz, K. G., Riebesell, U. & Schmittner, A. Simulated 21st century's increase in oceanic suboxia by CO 2-enhanced biotic carbon export. *Global Biogeochemical Cycles* **22**, 1–10 (2008).

7. Garcia, H. E. *et al.* World Ocean Atlas 2013. Vol. 3: Dissolved Oxygen, Apparent Oxygen Utilization, and Oxygen Saturation. S. Levitus, Ed.; A. Mishonov, Technical Ed. Tech. Rep., NOAA Atlas NESDIS 75 (2013).

8. Phipps, S. J. *et al.* The CSIRO Mk3L climate system model version 1.0 - Part 2: Response to external forcings. *Geoscientific Model Development* **5**, 649–682 (2012).

9. Phipps, S. J. *et al.* Paleoclimate Data-Model Comparison and the Role of Climate Forcings over the Past 1500 Years*. *Journal of Climate* **26**, 6915–6936 (2013).

10. Macdonald, A. M. The global ocean circulation: A hydrographic estimate and regional analysis. *Progress in Oceanography* **41**, 281–382 (1998).

11. Sloyan, B. M. & Rintoul, S. R. Circulation, Renewal, and Modification of Antarctic Mode and Intermediate Water*. *Journal of Physical Oceanography* **31**, 1005–1030 (2001).

12. Orsi, A. H., Smethie, W. M. & Bullister, J. L. On the total input of Antarctic waters to the deep ocean : A preliminary estimate from chlorofluorocarbon measurements. *Journal of Geophysical Research* **107**, 1–17 (2002).

13. Talley, L. D., Reid, J. L. & Robbins, P. E. Data-based meridional overturning streamfunctions for the global ocean. *Journal of Climate* **16**, 3213–3226 (2003).

14. Ganachaud, A. Large-scale mass transports, water mass formation, and diffusivities estimated from World Ocean Circulation Experiment (WOCE) hydrographic data. *Journal of Geophysical Research* **108**, 3213 (2003).

15. Lumpkin, R. & Speer, K. Global Ocean Meridional Overturning. *J. Phys. Oceanogr.* **37**, 2550–2562 (2007).

16. Mazloff, M. R., Heimbach, P. & Wunsch, C. An Eddy-Permitting Southern Ocean State Estimate. *Journal of Physical Oceanography* **40**, 880–899 (2010).

17. Iudicone, D. *et al.* Water masses as a unifying framework for understanding the Southern Ocean Carbon Cycle. *Biogeosciences* **8**, 1031–1052 (2011).

18. Buchanan, P. J., Matear, R. J., Chase, Z., Phipps, S. J. & Bindoff, N. L. Dynamic biological functioning important for simulating and stabilising ocean biogeochemistry. *Global Biogeochemical Cycles* (2018).

19. Cunningham, S. A., Alderson, S. G., King, B. A. & Brandon, M. A. Transport and variability of the Antarctic Circumpolar Current in Drake Passage. *Journal of Geophysical Research* **108**, 8084 (2003).

20. Donohue, K. A., Tracey, K. L., Watts, D. R., Chidichimo, M. P. & Chereskin, T. K. Mean Antarctic Circumpolar Current transport measured in Drake Passage. *Geophysical Research Letters* **43**, 11,760–11,767 (2016).

21. Martiny, A., Vrugt, J. & Lomas, M. Concentrations and ratios of particulate organic carbon, nitrogen, and phosphorus in the global ocean. *Scientific data* **1**, 140048 (2014).

22. Mahowald, N. M. *et al.* Atmospheric global dust cycle and iron inputs to the ocean. *Global Biogeochemical Cycles* **19**, n/a–n/a (2005).

23. Lambert, F. *et al.* Dust fluxes and iron fertilization in Holocene and Last Glacial Maximum climates. *Geophysical Research Letters* **42**, 6014–6023 (2015).

24. Taylor, K. E. Summarizing multiple aspects of model performance in a single diagram. *Journal of Geophysical Research* **106**, 7183 (2001).

25. Garcia, H. E. *et al.* World Ocean Atlas 2013. Vol. 4: Dissolved Inorganic Nutrients (phosphate, nitrate, silicate). S. Levitus, Ed.; A. Mishonov, Technical Ed. Tech. Rep. (2013).
