# Supplementary material for: Marine nitrogen fixers mediate a low latitude pathway for atmospheric CO2 drawdown
Source: Nat Commun. 2019 Oct 10;10:4611. doi: 10.1038/s41467-019-12549-z (PMC6787065; doi:10.1038/s41467-019-12549-z)
Supplement: Supplementary file 1 — Supplementary Information [file 41467_2019_12549_MOESM1_ESM.pdf]

Reviewers' comments:

Reviewer #1 (Remarks to the Author):

The authors model a new way of explaining a significant fraction of glacial CO2 drawdown, which until now, is still not fully understood. Most evidence points toward Fe fertilisation of the high latitudes (Southern Ocean and Northern Pacific Ocean) as a potential cause of CO2 sequestration during the glacial. However, previous model shows that such Fe fertilisation can explain only a fraction of the CO2 drawdown due to biological mechanisms. Their model calls upon Fe-induced stimulation of N2 fixation in the low latitudes (in particular, the Eastern Equatorial Pacific), and conservatively estimates that up to about half of the CO2 drawdown due to biological mechanisms can be explained.

The idea is a simple and elegant one, and I'm surprised that no one had thought of modeling this previously. In my mind, that is the sign of an excellent idea! It merits publication in Nature Communications. For the most part it is well written and well-organized. I have three concerns, however, before the manuscript can be published.

1) A schematic diagram like Figure 5 is important for the manuscript but, currently, Figure 5 is too busy and hard to decipher. I had to stare at it for long, before I started to understand it. Nominal latitudes would be useful. I don't understand the yellow and green circles, nor do I understand the tiny blobs with arrowheads in the deeper ocean. In the low-Fe scenario it seems as if most of the Fe deposition is in the central Pacific, whereas in the high-Fe scenario it seems as though most of the Fe deposition is in the eastern Pacific. Should there be any Fe deposition in the central Pacific in the low-Fe scenario? What is the teal and dark-green checkered area right over the equator—CO2 outgassing? It looks as though in the top view there is the same CO2 outgassing in both the low- and high-Fe scenarios. Wouldn't there be less CO2 outgassing in the high-Fe scenario? There are simply too many layers in the cross-sectional view and too many colors and symbols in the top view to make sense for the reader. The authors have to attempt to simplify this figure by making only the most important points. They then might further explain in the figure captions additional specific points.

2) For Figure 2, the authors should state in the figure caption that the glacial supply of Fe represents 500% of the modern supply of Fe. I believe the 500% number comes from the Lambert et al. (2015) reference. This number seems high to me. I was under the impression that the glacial dust supply was only double that of the modern dust supply to the tropical Pacific. To get to the 500% number, did the authors assume a different source of dust with different amounts of Fe, or different amounts of solubility of Fe? This has to be clarified. Also, just to be clear, the diminishing returns for CO2 sequestration from 500% to 2500% Fe supply, is related to increasing PO4 limitation. If this is correct, it might be mentioned in the figure caption just to emphasize the point.

3) The authors show a decent correlation between the glacial d15N of their model results with those of actual observations from the literature in Figure 4. One thing that is missing from the paper, however, and this is related to #2 above, is actual data concerning the glacial Fe supply. This is important because the Fe part of the story is just as critical as the N part of the story (after all, it is co-limitation by both Fe and N). And, there are observational studies that the authors should include which do show that the Fe supply is doubled during the glacial timescales (summarized in Costa et al., 2017) and, even more importantly, millennial timescales (Loveley et al., 2017). The latter paper also shows an increase in the extent of oceanic hypoxia on glacial and millennial timescales consistent with the hypothesis put forward in the manuscript.

References
Costa, K. M. et al. (2017) Productivity patterns in the equatorial Pacific over the last 30,000 years. Glob. Biogeochem. Cycles 31, 850–865.

Loveley, M. R. et al. (2017) Millennial-scale iron fertilization of the eastern equatorial Pacific over the past 100,000 years. Nat. Geosci. 10, 760–764.

Reviewer #2 (Remarks to the Author):

Review of "Marine nitrogen fixers mediate a low latitude pathway for atmospheric CO2 drawdown" by Buchanan et al. for Nature Communications

Understanding the mechanisms responsible for the drawdown in atmospheric CO2 concentrations during the glacial period is a key issue in paleoclimatology. After decades of research, we still do not fully understand these mechanisms. Most often, processes associated with the Southern Ocean are invoked – but these processes are likely not enough to account for the full amount of the drawdown. The paper by Buchanan et al. represents a key step forward in understanding how the low latitudes may be a key player through dust-borne iron stimulation of N2 fixation. They provide modeling results suggesting that this low latitude pathway can account for an additional 7-16ppm of CO2 drawdown in the glacial ocean. Overall, I find this to be a well written paper, and could be a potentially transformative finding to understand glacial-interglacial CO2 dynamics. I have only a couple minor comments that I think could be addressed before publication.

(1) Lack of discussion of recent studies for low latitude Fe-fertilization: I think one key point that is missing from this paper are citations to articles showing actual evidence for dust borne iron fertilization in the glacial ocean. One example is Loveley et al. 2017 (Nature Geoscience). Similarly, the authors use the Mahowald et al. 2005 reference for dust supply estimates, but a new study, Kienast et al. 2016 (Global Biogeochemical Cycles) which looked at thorium derived dust fluxes should also be included.
(2) The authors state the parameters for the cycles included in the model on page 2, line 33 – but it seems that Si is missing. From my understanding, silicon can be a major limiting nutrient for diatom growth, so I am wondering what, if any, effect this would have on the maximum amount of productivity that the low latitudes can sustain. Similarly, it would be helpful to include a discussion on the dominant phytoplankton communities in these Fe-limited regions and how the community might be altered under glacial conditions or conditions where Fe is no longer limiting. What impact does changes in the phytoplankton community have on the N2 fixation model?

Reviewer #3 (Remarks to the Author):

The study examines the potential for stimulation of N2 fixation by atmospheric Fe deposition to contribute to the glacial drawdown of atmospheric CO2. It encompasses a large set of well-designed model experiments, and attributes significant CO2 drawdown to this process. The paper is well-written and contains quite interesting and significant results. They make a strong case for this mechanism as a contributor to the glacial CO2 drawdown. I strongly support publication after some minor clarifications and modifications. Detailed suggestions are provided below.

Abstract
I'm not sure about the wording of "stronger spatial coupling…". Most of the increased N fixation and carbon export is not directly above the denitrification zones (Fig 1). Directly above the denitrification zone is what I would consider "coupled" (see also Wang et al. reference below). Maybe re-phrase as a "weaker spatial de-coupling.." ….

Line 19, suggest removing word anaemic

Lines 13-14. These lines are somewhat confusing. As written, it implies that across the low latitudes there is co-limitation by Fe and N. However, the references cited refer to this co-limitation along the boundaries where Fe-limited and N-limited regions meet. The biggest response to the Fe deposition at low latitudes is in such a boundary region in the Pacific. These points need to be spelled out more clearly.

Lines 13-26. The two references listed below are relevant for this discussion. The inverse model results of Wang et al. are quite relevant for many of the ideas discussed here, including both the N fixation and the variable N/P stoichiometry, and are generally supportive of the N/P ratios and N fixation in the base model used in this study.

2006. Moore, JK, SC Doney, Lindsay, K, Mahowald, N, 2006. Nitrogen fixation amplifies the ocean biogeochemical response to decadal timescale variations in mineral dust deposition. Tellus 58B, 560-572.

2019. Wang, W.L., Moore, J.K., Martiny, A.C., F.W. Primeau (2019). Convergent estimates of marine nitrogen fixation. Nature, 566, 205-211, https://doi.org/10.1038/s41586-019-0911-2.

Page 8
The C/P of the diazotrophs is discussed and manipulated here, but there is no mention of the C/P of the non-diazotrophs (although there is a figure in the supp.). What are the C/P of the non-diazotrophs in this tropical Pacific region? Do these change dynamically with the environment, if so how does this impact the carbon export/CO2 drawdown?

Page 10, bottom
This balance and intensity would also be sensitive to bottom water O2 concentrations.

Page 13, bottom
I acknowledge it is tricky to isolate certain regions for experiments like this with a global model. But I’m concerned making the high latitudes “nearly Fe replete” might have impacted the results by changing the thermocline nutrient concentrations in the base model. Increasing SO Fe inputs should stimulate the biological pump strongly in that region and decrease the northward flow of nutrients into the low latitude thermocline. If these subsurface nutrient concentrations are too low, the model might be overly sensitive to variations in Fe deposition and N fixation.

There is little discussion of the glacial sedimentary records regarding export production.

In the no N fixers experiment, there is little carbon accumulation in the oceans. Is this because big increases in CO2 uptake in the upwelling/HNLC area are balanced by decreased productivity and CO2 outgassing (as N-limitaion intensifies) in areas laterally downstream? If so, this is worth discussing and laying out more clearly.

Reviewers' comments:

Reviewer #1 (Remarks to the Author):

The authors model a new way of explaining a significant fraction of glacial CO2 drawdown, which until now, is still not fully understood. Most evidence points toward Fe fertilisation of the high latitudes (Southern Ocean and Northern Pacific Ocean) as a potential cause of CO2 sequestration during the glacial. However, previous model shows that such Fe fertilisation can explain only a fraction of the CO2 drawdown due to biological mechanisms. Their model calls upon Fe-induced stimulation of N2 fixation in the low latitudes (in particular, the Eastern Equatorial Pacific), and conservatively estimates that up to about half of the CO2 drawdown due to biological mechanisms can be explained.

The idea is a simple and elegant one, and I'm surprised that no one had thought of modeling this previously. In my mind, that is the sign of an excellent idea! It merits publication in Nature Communications. For the most part it is well written and well-organized. I have three concerns, however, before the manuscript can be published.

1) A schematic diagram like Figure 5 is important for the manuscript but, currently, Figure 5 is too busy and hard to decipher. I had to stare at it for long, before I started to understand it. Nominal latitudes would be useful. I don't understand the yellow and green circles, nor do I understand the tiny blobs with arrowheads in the deeper ocean. In the low-Fe scenario it seems as if most of the Fe deposition is in the central Pacific, whereas in the high-Fe scenario it seems as though most of the Fe deposition is in the eastern Pacific. Should there be any Fe deposition in the central Pacific in the low-Fe scenario? What is the teal and dark-green checkered area right over the equator—CO2 outgassing? It looks as though in the top view there is the same CO2 outgassing in both the low- and high-Fe scenarios. Wouldn't there be less CO2 outgassing in the high-Fe scenario? There are simply too many layers in the cross-sectional view and too many colors and symbols in the top view to make sense for the reader. The authors have to attempt to simplify this figure by making only the most important points. They then might further explain in the figure captions additional specific points.

ACTIONED.

The schematic is redrawn and additional explanation is given in the caption. We have removed a few unnecessary components, specifically the CO_2 outgassing layer, dust layer and small squiggly C export arrows. We have also included nominal latitudes and longitudes.

2) For Figure 2, the authors should state in the figure caption that the glacial supply of Fe represents 500% of the modern supply of Fe. I believe the 500% number comes from the Lambert et al. (2015) reference. This number seems high to me. I was under the impression that the glacial dust supply was only double that of the modern dust supply to the tropical Pacific. To get to the 500% number, did the authors assume a different source of dust with different amounts of Fe, or different amounts of solubility of Fe? This has to be clarified. Also, just to be clear, the diminishing returns for CO2 sequestration from 500% to 2500% Fe supply, is related to increasing PO4 limitation. If this is correct, it might be mentioned in the figure caption just to emphasize the point.

ACTIONED.

500% has been added to both the caption and within the figure legend.

500% is indeed high for the tropical pacific, yet this number represents the global integrated increase. Locally, the tropical Pacific receives less Fe than other regions, and it is clear from Figure S2 that this increase is on the order of 2-3 fold. To make this clear to the reader, we have mentioned this upfront in the text and also added an additional panel to Supplementary Figure 2, which shows the regional increase in Fe.

Diminishing returns of CO2 sequestration with additional Fe is now mentioned in the caption.

3) The authors show a decent correlation between the glacial d15N of their model results with those of actual observations from the literature in Figure 4. One thing that is missing from the paper, however, and this is related to #2 above, is actual data concerning the glacial Fe supply. This is important because the Fe part of the story is just as critical as the N part of the story (after all, it is co-limitation by both Fe and N).

ACTIONED.

The Loveley et al. (2017) and Kienast et al. (2016) papers have been referenced to address this. Both suggest an increase in atmospheric dust loading of roughly double in the tropical Pacific during the glacial periods. We have further added an addition panel to Supplementary Figure 2 to show the difference between the glacial and modern Fe deposition fields, which shows only a doubling-tripling of Fe supply to the surface ocean under the glacial scenario.

And, there are observational studies that the authors should include which do show that the Fe supply is doubled during the glacial timescales (summarized in Costa et al., 2017) and, even more importantly, millennial timescales (Loveley et al., 2017). The latter paper also shows an increase in the extent of oceanic hypoxia on glacial and millennial timescales consistent with the hypothesis put forward in the manuscript.

References

Costa, K. M. et al. (2017) Productivity patterns in the equatorial Pacific over the last 30,000 years. Glob. Biogeochem. Cycles 31, 850–865.

Loveley, M. R. et al. (2017) Millennial-scale iron fertilization of the eastern equatorial Pacific over the past 100,000 years. Nat. Geosci. 10, 760–764.

ACTIONED.

Our study supports Loveley et al. (2017) and Marcantonio et al. (2019) by providing a mechanism to explain how iron fertilization could enable CO_2 drawdown in the lower latitudes. Specifically, that:

1. **A simulated, positive connection between dust and xsBa flux to the sediments via a vertical expansion of suboxic zones (as Loveley et al. (2017) and Hoogakker et al. (2018) suggest).**
2. **the supply of iron to the surface ocean via aeolian sources is likely to have strong effects on the N_2 fixers based on many prior studies, and that the resulting supply of both Fe and NO_3 would alleviate NO_3 and Fe limitation in the lower latitudes, thereby increasing nutrient utilisation as shown previously (Pichevin et al., 2009) while explaining apparent inconsistencies in delta15N data (Costa et al., 2016).**
3. **A mechanism involving C:P variability and effective injection of c_{org} in suboxic zones to permanently draw CO_2 into the ocean via the low latitudes, thereby refuting the third argument of Jacobel et al. (2019).**

These points have been integrated within the manuscript with an additional discussion point, where we also suggest a reconciliation to the contradiction of low particle export (Costa et al. 2017 GBC) with greater organic carbon burial (Cartapanis et al., 2016 Nature Communications).

(Pichevin et al., 2009) **– Enhanced carbon pump inferred from relaxation of nutrient limitation in the glacial ocean.**

(Kienast et al., 2016) **– Tracing dust input to the global ocean using thorium isotopes in marine sediments: ThoroMap. *Global biogeochemical cycles.***

(Loveley et al., 2017) – Millennial-scale iron fertilization of the eastern equatorial Pacific over the past 100,000 years.

(Jacobel et al., 2019) – No evidence for equatorial Pacific dust fertilization.

(Marcantonio et al., 2019) **– Reply to: No evidence for equatorial Pacific dust fertilization.**

Reviewer #2 (Remarks to the Author):

Review of “Marine nitrogen fixers mediate a low latitude pathway for atmospheric CO2 drawdown” by Buchanan et al. for Nature Communications

Understanding the mechanisms responsible for the drawdown in atmospheric CO2 concentrations during the glacial period is a key issue in paleoclimatology. After decades of research, we still do not fully understand these mechanisms. Most often, processes associated with the Southern Ocean are invoked – but these processes are likely not enough to account for the full amount of the drawdown. The paper by Buchanan et al. represents a key step forward in understanding how the low latitudes may be a key player through dust-borne iron stimulation of N2 fixation. They provide modeling results suggesting that this low latitude pathway can account for an additional 7-16ppm of CO2 drawdown in the glacial ocean. Overall, I find this to be a well written paper, and could be a potentially transformative finding to understand glacial-interglacial CO2 dynamics. I have only a couple minor comments that I think could be addressed before publication.

(1) Lack of discussion of recent studies for low latitude Fe-fertilization: I think one key point that is missing from this paper are citations to articles showing actual evidence for dust borne iron fertilization in the glacial ocean. One example is Loveley et al. 2017 (Nature Geoscience). Similarly, the authors use the Mahowald et al. 2005 reference for dust supply estimates, but a new study, Kienast et al. 2016 (Global Biogeochemical Cycles) which looked at thorium derived dust fluxes should also be included.

ACTIONED.

The Lovely and Kienast papers have been included in the manuscript. Both show roughly a 2-3 fold increase in dust supply to the tropical Pacific, which matches our the Lambert dust deposition field which gives a doubling of dust in the tropics. Please see Supplementary Figure 2 for this, as we have added another panel to clearly show the change in Fe deposition in a regional sense.

(2) The authors state the parameters for the cycles included in the model on page 2, line 33 – but it seems that Si is missing. From my understanding, silicon can be a major limiting nutrient for diatom growth, so I am wondering what, if any, effect this would have on the maximum amount of productivity that the low latitudes can sustain. Similarly, it would be helpful to include a discussion on the dominant phytoplankton communities in these Fe-limited regions and how the community might be altered under glacial conditions or conditions where Fe is no longer limiting. What impact does changes in the phytoplankton community have on the N2 fixation model?

ANSWER TO QUESTION.

It's true that our model does not include the Si cycle. Our model includes only three forms of phytoplankton: a general group, nitrogen fixers and calcifiers. Hence, the response is limited to these groups. Diatoms are therefore not explicitly modelled but their functional role is included in the general group. We do not expect our major result, being the link between N_2-fixation and CO_2 drawdown, to change with the inclusion of a Si cycle and diatoms, but significant new model development is required to test this hypothesis.

Likewise, a major theme of this work is the impact of Fe fertilisation on the phytoplankton community, namely the rise in importance of N2 fixers, and because our model has only 3 phytoplankton groups we cannot say anything more about the alteration of community structure from this work. We agree that this is an important topic and it's one we hope to address in the future using a more complex version of the model.

Reviewer #3 (Remarks to the Author):

The study examines the potential for stimulation of N2 fixation by atmospheric Fe deposition to contribute to the glacial drawdown of atmospheric CO2. It encompasses a large set of well-designed model experiments, and attributes significant CO2 drawdown to this process. The paper is well-written and contains quite interesting and significant results. They make a strong case for this mechanism as a contributor to the glacial CO2 drawdown. I strongly support publication after some minor clarifications and modifications. Detailed suggestions are provided below.

Abstract
I'm not sure about the wording of "stronger spatial coupling…". Most of the increased N fixation and carbon export is not directly above the denitrification zones (Fig 1). Directly above the denitrification zone is what I would consider "coupled" (see also Wang et al. reference below). Maybe re-phrase as a "weaker spatial de-coupling.." ….

ACTIONED.

We have replaced the words "stronger spatial coupling" with "closer coupling". Hopefully this is more intuitive for the reader.

The suggested alternative of a "weaker spatial de-coupling" by the reviewer is a double negative and we believe is not immediately intuitive, thus possibly making the results less interpretable without re-reading a sentence or two.

Line 19, suggest removing word anaemic

ACTIONED.

Noted. Replaced with "Fe-limited".

Lines 13-14. These lines are somewhat confusing. As written, it implies that across the low latitudes there is co-limitation by Fe and N. However, the references cited refer to this co-limitation along the boundaries where Fe-limited and N-limited regions meet. The biggest response to the Fe deposition at low latitudes is in such a boundary region in the Pacific. These points need to be spelled out more clearly.

ACTIONED.

We have re-written paragraph 3 of the Introduction to make these points clearer. We agree with the reviewer here that it was not clear enough in the initial version. To ensure clarity, we have explained that there are three regimes of limitation: NO3 lim, Fe lim and NO3-Fe co-lim. As all involve NO3 and Fe as the limiting nutrients, we have then lead into how an Fe-induced stimulation of nitrogen fixation can simultaneously alleviate all of these regimes. Please see paragraph 3.

Lines 13-26. The two references listed below are relevant for this discussion. The inverse model results of Wang et al. are quite relevant for many of the ideas discussed here, including both the N fixation and the variable N/P stoichiometry, and are generally supportive of the N/P ratios and N fixation in the base model used in this study.

2006. Moore, JK, SC Doney, Lindsay, K, Mahowald, N, 2006. Nitrogen fixation amplifies the ocean biogeochemical response to decadal timescale variations in mineral dust deposition. Tellus 58B, 560-572.

2019. Wang, W.L., Moore, J.K., Martiny, A.C., F.W. Primeau (2019). Convergent estimates of marine nitrogen fixation. Nature, 566, 205-211, https://doi.org/10.1038/s41586-019-0911-2.

ACTIONED.

Wang et al., 2019 citation included as a reference to show how N2 fixation and denitrification are decoupled and that N2 fixation supports C export where it exists.

Moore et al., 2006 referenced up front in the third paragraph of the Introduction.

Page 8

The C/P of the diazotrophs is discussed and manipulated here, but there is no mention of the C/P of the non-diazotrophs (although there is a figure in the supp.). What are the C/P of the non-diazotrophs in this tropical Pacific region? Do these change dynamically with the environment, if so how does this impact the carbon export/CO2 drawdown?

ACTIONED.

We have included an extra sentence within this section to note that the increase in C storage due to N supply also included the increase in C:P of the phytoplankton community. We additionally point the reader towards Fig.1, reminding them of the increase in tropical Pacific C:P.

Page 10, bottom

This balance and intensity would also be sensitive to bottom water O2 concentrations.

ACTIONED.

We have made this clearer by including a reference to the findings of Anderson et al., 2019, Global Biogeochemical Cycles, and mentioning that the deoxygenation of bottom waters would have stimulated denitrification.

Anderson, R. F. et al. Deep-Sea Oxygen Depletion and Ocean Carbon Sequestration During the Last Ice Age. Global Biogeochemical Cycles 33, 301–317 (2019).

Page 13, bottom

I acknowledge it is tricky to isolate certain regions for experiments like this with a global model. But

I'm concerned making the high latitudes "nearly Fe replete" might have impacted the results by changing the thermocline nutrient concentrations in the base model. Increasing SO Fe inputs should stimulate the biological pump strongly in that region and decrease the northward flow of nutrients into the low latitude thermocline. If these subsurface nutrient concentrations are too low, the model might be overly sensitive to variations in Fe deposition and N fixation.

ANSWER TO QUESTION.

The reviewer is concerned that making the higher latitudes "nearly Fe-replete" in the base model might have created unrealistically high rates of nutrient utilisation in the high latitudes, thereby reducing the nutrient supply to the lower latitudes via subducting mode waters. The loss of nutrient supply might have made lower latitude C export more sensitive to gains in N_2 fixation than if more NO_3 and PO_4 were being supplied to lower latitude thermoclines.

As our study assessed the *change* in CO_2 drawdown between low and high Fe supply scenarios, we would actually expect this *change* to be less in our experiments than if the HNLC regions were more sensitive to Fe supply. Our estimated CO_2 drawdown via the low latitudes may in fact be an underestimate.

To explain more, imagine that the HNLC regions were strongly Fe-limited under the modern Aeolian Fe supply. In this case, the addition of Fe would have a very strong effect on nutrient consumption in the high latitudes and subsequent export of nutrients in mode waters from the high to low latitudes. Thermocline concentrations of PO_4 and NO_3 would decline as more Fe stimulated export production in HNLC regions. The nutrient supply to low latitude thermoclines would therefore reduce. In this case, global N_2 fixation would be weaker than in our "modern Fe supply" experiments because higher concentrations of NO_3 would sit within the global thermocline. The introduction of more Fe from above would then simultaneously increase Fe while decreasing thermocline NO_3, strongly favouring N_2 fixers. Gains in the global N_2 fixation rate would be larger than what is recorded in our experiments.

As one final note, our sensitivity tests using different circulations show that CO_2 drawdown is sensitive to the relative amounts of NO_3 and PO_4 that are supplied to the tropics. In effect, this could also be viewed as a sensitivity to how much NO_3 and PO_4 are exported out of the Southern Ocean via mode/intermediate waters.

ACTION.

An additional paragraph has been added to the discussion (3rd paragraph) that acknowledges that the high latitudes are insensitive to the changes in Fe fertilisation. We mention that no change in the properties of intermediate and mode waters is integrated within our results.

POSSIBLE ACTION.

If the reviewer and editor think necessary, we could address this more completely as an extra paragraph within the discussion, speculating how changes in the properties of southern ocean mode and intermediate waters might amplify our results. However, at the moment we feel that the addition of such a paragraph might dilute the discussion, which currently:

1. **Confirms that N_2 fixation is an integral part of the C cycle on glacial timescales.**

2. **Offers an alternative interpretation of the glacial d15N records and particle export rates in the tropical pacific to show that CO_2 drawdown is possible here.**
3. **Proposes that N_2 fixation today is not operating at its full capacity, which means the biological pump is operating below is full potential.**

There is little discussion of the glacial sedimentary records regarding export production.

ACTIONED.

A brief paragraph in the discussion has been added to address the debate regarding equatorial pacific export production, specifically the seemingly irreconcilable observations of low particle export (Costa et al., 2017, GBC) with greater organic carbon export.

Costa, K. M. et al. (2017) Productivity patterns in the equatorial Pacific over the last 30,000 years. Glob. Biogeochem. Cycles 31, 850–865.

In the no N fixers experiment, there is little carbon accumulation in the oceans. Is this because big increases in CO2 uptake in the upwelling/HNLC area are balanced by decreased productivity and CO2 outgassing (as N-limitaion intensifies) in areas laterally downstream? If so, this is worth discussing and laying out more clearly.

ANSWER TO QUESTION.

The reviewers explanation is correct given our results and it is a useful interpretation of the simulations. What this says is that aeolian Fe supply has a limited capacity to increase carbon storage in the ocean (via the low latitude pathway we present) unless NO_3 supply also increases through N_2 fixation.

While these insights are true, we feel that the inability to effect CO_2 drawdown through only Fe supply (without N2 fixers) is likely a consequence of our choice to make the HNLC regions *weakly* Fe-limited. There is simply little response in terms of C export or CO_2 drawdown because there is actually little response in the HNLC regions when Fe is added. Fe limitation quickly gives way to light limitation and there is no *big increase* in CO_2 uptake. In a global sense, we are therefore hesitant to say that Fe fertilisation without gains in N_2 fixation could not draw CO_2 into the ocean.

ACTION.

We have added a paragraph to the discussion to address this point. The new paragraph is the third in the discussion and makes it clear that our results should be interpreted as an isolated low latitude response.

REVIEWERS' COMMENTS:

Reviewer #1 (Remarks to the Author):

The authors have done an excellent job of revising their manuscript vis-a-vis ALL of the constructive reviews.

Reviewer #2 (Remarks to the Author):

Thank you to the authors for providing a thorough response to my concerns. All of my concerns have been addressed and at this time I support publication of the manuscript in Nature Communications.

Reviewer #3 (Remarks to the Author):

I have read the revised version of this manuscript, and I am satisfied that all of my concerns and those of the other reviewers have been addressed. I think this is a very strong, important paper that should be published. In sum, I strongly support publishing the revised manuscript.

REVIEWERS' COMMENTS:

Reviewer #1 (Remarks to the Author):

The authors have done an excellent job of revising their manuscript vis-a-vis ALL of the constructive reviews.

Reviewer #2 (Remarks to the Author):

Thank you to the authors for providing a thorough response to my concerns. All of my concerns have been addressed and at this time I support publication of the manuscript in Nature Communications.

Reviewer #3 (Remarks to the Author):

I have read the revised version of this manuscript, and I am satisfied that all of my concerns and those of the other reviewers have been addressed. I think this is a very strong, important paper that should be published. In sum, I strongly support publishing the revised manuscript.

As there are no further issues from the three reviewers, we have provided a largely unaltered manuscript to the editor. The only alterations are those related to formatting requirements and suggestions by the editor.

Thanks to all reviewers and the editor during this process.
