# Supplementary material for: Marine nitrogen fixers mediate a low latitude pathway for atmospheric CO2 drawdown
Source: Nat Commun. 2019 Oct 10;10:4611. doi: 10.1038/s41467-019-12549-z (PMC6787065; doi:10.1038/s41467-019-12549-z)
Supplement: Supplementary file 2 — Peer Review File [file 41467_2019_12549_MOESM2_ESM.pdf]

Description of Additional Supplementary Files

File Name: Supplementary Data 1

Description: Glacial-interglacial values of delta15N within organic matter from marine sedimentary records.
